# Romidepsin-CHOEP followed by high-dose chemotherapy and stem-cell transplantation in untreated Peripheral T-Cell Lymphoma: results of the PTCL13 phase Ib/II study

**DOI:** 10.1038/s41375-022-01780-1

**Published:** 2023-01-18

**Authors:** Annalisa Chiappella, Anna Dodero, Andrea Evangelista, Alessandro Re, Lorella Orsucci, Sara Veronica Usai, Claudia Castellino, Vittorio Stefoni, Antonio Pinto, Manuela Zanni, Rosanna Ciancia, Chiara Ghiggi, Francesca Gaia Rossi, Annalisa Arcari, Fiorella Ilariucci, Vittorio Ruggero Zilioli, Leonardo Flenghi, Melania Celli, Stefano Volpetti, Fabio Benedetti, Filippo Ballerini, Gerardo Musuraca, Riccardo Bruna, Caterina Patti, Francesco Leonardi, Luca Arcaini, Massimo Magagnoli, Federica Cavallo, Anisa Bermema, Alessandra Tucci, Carola Boccomini, Giovannino Ciccone, Cristiana Carniti, Stefano Aldo Pileri, Paolo Corradini

**Affiliations:** 1 Division of Hematology and Stem Cell Transplantation, Fondazione IRCCS Istituto Nazionale dei Tumori, Milano, Italy; 2 Unit of Clinical Epidemiology, Azienda Ospedaliera e Universitaria Città della Salute e della Scienza and CPO Piemonte, Torino, Italy; 3 Hematology Division, ASST Spedali Civili di Brescia, Brescia, Italy; 4 Division of Hematology, Azienda Ospedaliera e Universitaria Città della Salute e della Scienza, Torino, Italy; 5 Hematology Unit, Ospedale Oncologico Armando Businco, Cagliari, Italy; 6 Division of Hematology, Azienda Ospedaliera S. Croce e Carle, Cuneo, Italy; 7 Institute of Hematology “Seràgnoli”, University of Bologna, Bologna, Italy; 8 Hematology-Oncology & Stem Cell Transplantation Unit, Istituto Nazionale Tumori, Fondazione G. Pascale, IRCCS, Napoli, Italy; 9 Division of Hematology, Azienda Ospedaliera Santi Antonio e Biagio e Cesare Arrigo, Alessandria, Italy; 10 Onco-hematology and Stem Cell Transplantation and Cellular Therapies, Centro di Riferimento Oncologico di Aviano (CRO) IRCCS, Aviano, Italy; 11 Unit of Hematology and Cellular therapies, IRCCS Ospedale Policlinico San Martino, Genova, Italy; 12 Division of Hematology, Fondazione IRCCS Cà Granda, OM Policlinico, Milano, Italy; 13 Hematology Unit, Ospedale Guglielmo da Saliceto, Piacenza, Italy; 14 Hematology, Azienda USL-IRCCS di Reggio Emilia, Reggio Emilia, Italy; 15 Division of Hematology, ASST Grande Ospedale Metropolitano Niguarda, Milano, Italy; 16 Hematology, Azienda Ospedaliera di Perugia, Perugia, Italy; 17 Hematology, Ospedale degli Infermi, Rimini, Italy; 18 Clinic of Hematology, Presidio Ospedaliero Universitario “Santa Maria della Misericordia” di Udine, ASUFC, Udine, Italy; 19 Hematology and Stem Cell Transplantation, Azienda Ospedaliera Universitaria di Verona, Verona, Italy; 20 Hematology, IRCCS Ospedale Policlinico San Martino, Genova, Italy; 21 Hematology unit, IRCCS Istituto Romagnolo per lo Studio dei Tumori (IRST) “Dino Amadori”, Meldola, Italy; 22 Division of Hematology, Ospedale Maggiore Della Carità, Novara, Italy; 23 Division of Onco-Hematology, Azienda Villa Sofia Cervello, Palermo, Italy; 24 Hematology and CTMO, Azienda Ospedaliera-Universitaria di Parma, Parma, Italy; 25 Department of Molecular Medicine, University of Pavia & Division of Hematology, Fondazione IRCCS Policlinico San Matteo, Pavia, Italy; 26 Department of Oncology and Hematology, Humanitas Cancer Center, IRCCS Humanitas Research Hospital, Rozzano, Italy; 27 Division of Hematology, Department of Molecular Biotechnologies and Health Sciences, University of Torino/Azienda Ospedaliera e Universitaria Città della Salute e della Scienza, Torino, Italy; 28 Laboratory of Hematology, Division of Hematology and Stem Cell Transplantation, Fondazione IRCCS Istituto Nazionale dei Tumori, Milano, Italy; 29 Division of Haematopathology, European Institute of Oncology IRCCS, Milano, Italy; 30 Chair of Hematology, University of Milano, Milano, Italy

**Keywords:** T-cell lymphoma, Stem-cell research

## Abstract

The standard treatment for young patients with untreated PTCLs is based on anthracycline containing-regimens followed by high-dose-chemotherapy and stem-cell-transplantation (HDT + SCT), but only 40% of them can be cured. Romidepsin, a histone-deacetylase inhibitor, showed promising activity in relapsed PTCLs; in first line, Romidepsin was added with CHOP. We designed a study combining romidepsin and CHOEP as induction before HDT + auto-SCT in untreated PTCLs (PTCL-NOS, AITL/THF, ALK-ALCL), aged 18–65 years. A phase Ib/II trial was conducted to define the maximum tolerated dose (MTD) of Ro-CHOEP, and to assess efficacy and safety of 6 Ro-CHOEP as induction before HDT. The study hypothesis was to achieve a 18-month PFS of 70%. Twenty-one patients were enrolled into phase Ib; 7 dose-limiting toxicities were observed, that led to define the MTD at 14 mg/ms. Eighty-six patients were included in the phase II. At a median follow-up of 28 months, the 18-month PFS was 46.2% (95%CI:35.0–56.7), and the 18-month overall survival was 73.1% (95%CI:61.6–81.7). The overall response after induction was 71%, with 62% CRs. No unexpected toxicities were reported. The primary endpoint was not met; therefore, the enrollment was stopped at a planned interim analysis. The addition of romidepsin to CHOEP did not improve the PFS of untreated PTCL patients.

## Introduction

Peripheral T-cell lymphomas (PTCLs) represent 10–15% of all non-Hodgkin lymphomas, are composed by several histological subtypes with different prognoses. With the exception of anaplastic lymphoma kinase (ALK)-positive anaplastic large cell lymphoma (ALK-positive ALCL), their outcome is still unsatisfactory [1].

Considering the most frequent entities, such as peripheral T-cell lymphoma not otherwise specified (PTCL-NOS), angioimmunoblastic T-cell lymphoma (AITL), and ALK-negative ALCL, the common first-line treatment for advanced-stage disease is based on cyclophosphamide, doxorubicin, vincristine, prednisone, (CHOP) with or without etoposide (CHOEP), usually followed by consolidation with high dose chemotherapy (HDT) and autologous stem cell transplantation (auto-SCT), in patients aged less than 65 years [2–4].

The intensification with auto-SCT in patient achieving first remission after induction, has been tested in several phase 2 studies in which ~30% of patients did not reach the transplant phase due to refractory disease [5, 6]. D’Amore et al. [7] reported the NLG-T-01 trial results; 160 patients were enrolled to receive 6 courses of CHOEP and 115 (72%) underwent the final auto-SCT. Complete response (CR) was achieved in 90 (56%) patients, and treatment-related mortality (TRM) was 4%. At 60.5 months of median follow-up, 5-year OS and PFS were 51% (95% CI, 43% to 59%) and 44% (95% CI, 36% to 52%), respectively. In the Italian PTCL06 trial [8], we reported the outcome of 61 young patients treated with a combination of a biological agent, alemtuzumab, with two courses of CHOP, followed by high-dose chemotherapy and a final consolidation with autologous or allogeneic SCT based upon donor availability. With this approach, 62% of the patients were able to underwent the transplant phase and, at a median follow-up of 40 months, the 4-year OS and PFS were 49% and 44%, respectively. Similar results were also confirmed by real-world experiences [9, 10].

Romidepsin, a histone deacetylase (HDAC) inhibitor, was tested in relapsed or refractory PTCLs, showing a promising activity, with objective responses ranging from 25 to 38%. The safety profile was acceptable, and the most frequent toxicities were hematological [11, 12].

The Lymphoma Study Association conducted a phase Ib-II dose escalation and expansion study evaluating the combination of romidepsin with CHOP in untreated PTCLs. Thirty-seven patients were enrolled and received eight courses of CHOP in association with varying doses of romidepsin (8–10–12 mg/ms) on days 1 and 8; based on a 3 + 3 design, the investigators chose romidepsin at 12 mg/ms as the recommended dose for phase II trials [13]. The same combination was further applied in a randomized trial testing CHOP versus Ro-CHOP in 421 adult patients with untreated PTCLs; the results of the randomized study showed no advantage in adding romidepsin to CHOP versus Ro-CHOP, with a median PFS of 12 months (95% CI, 9.0–25.8) versus 10.2 months (95% CI, 7.4–13.2), hazard ratio of 0.81 (*p* = 0.096) [14].

On these premises, we designed a phase Ib/II trial (PTCL13) in young patients with newly diagnosed PTCL to define the maximum tolerated dose (MTD) and to evaluate the safety, tolerability and efficacy of romidepsin in addition to CHOEP, in a program containing a final consolidation with SCT. Here, we report the final analysis of the phase Ib and the phase II part of PTCL13 trial.

## Materials/subjects and methods

### Study design and participants

PTCL13 was an open-label, multicenter phase Ib/II trial, that was conducted in 26 Italian centers of the Fondazione Italiana Linfomi. Eligible patients were aged 18–65 years and had untreated PTCL-NOS, angioimmunoblastic/T follicular helper (AITL/THF) and ALK-negative ALCL without central nervous system disease. A detailed list of inclusion and exclusion criteria is provided in the supplementary materials.

The trial was conducted in accordance with the Declaration of Helsinki and good clinical practice guidelines. Ethical approval was obtained by institutional review boards at each site. All participants provided written informed consent. This trial was registered with ClinicalTrials.gov, number NCT02223208.

### Procedures

The histological diagnosis was performed locally and then centrally reviewed by an expert hematopathologist (S.A.P), according to the 2017 edition of the WHO classification of lymphoid neoplasms [1]. Disease evaluation was performed according to Lugano criteria 2014 [15].

The treatment consisted of an induction phase and a consolidation phase with HDT and transplantation, Supplementary Fig. 1.

During induction, patients received three courses of Ro-CHOEP, followed by an interim evaluation with CT-scan, BM biopsy (if positive at baseline), and PET (recommended, not mandatory); patients in CR or partial response (PR) received three additional Ro-CHOEP courses. At the end of induction, patients in CR received one DHAP course (cisplatin, cytarabine, dexamethasone) to mobilize peripheral blood stem cells followed by auto-SCT, whereas the patients in PR with a donor available proceeded directly (DHAP not mandatory) to allo-SCT; patients in PR without a donor available, proceeded to DHAP and auto-SCT. Treatment failures at any time during the study, proceeded to salvage treatment, according to each institutional policy.

After MTD definition, romidepsin 14 mg/ms was administered by a 4-hours infusion, on days 1 and 8.

In patients eligible for auto-SCT, standard conditioning regimens with intravenous BCNU or Fotemustine or CCNU, etoposide, cytarabine, melphalan (BEAM or FEAM or CEAM) were allowed.

In patients in PR after induction, allo-SCT was performed if a HLA-identical or one antigen mismatched donor was available. In allo-SCT, a conditioning regimen based on intravenous thiotepa, cyclophosphamide, and fludarabine was recommended, with cyclosporine and short-course methotrexate as graft versus host disease (GvHD) prophylaxis [16].

### Outcomes

#### Phase Ib

The primary objective of phase Ib was to define the MTD of romidepsin in addition to CHOEP; the secondary objective was to assess the feasibility of Ro-CHOEP in a program including high-dose chemotherapy and transplantation.

The primary endpoint was to determine the incidence of dose-limiting toxicity (DLT) of romidepsin, considering as maximum dose the one causing any grade ≥3 non-hematologic toxicity or a delay >15 days of planned cycle, during the first two cycles according to the definitions of NCI Common Terminology Criteria for Adverse Events (CTCAE), version 4.0 (2009).

Secondary endpoints were to evaluate the proportion of patients reaching SCT and the overall response rate (ORR) at the end of the induction with Ro-CHOEP.

#### Phase II

The primary objective of phase II was to evaluate the activity in term of PFS and to confirm the safety of Ro-CHOEP.

The primary endpoint was the PFS, calculated from the date of enrollment to the date of disease progression, relapse, or death from any cause.

The secondary endpoints included the ORR and the CR, after Ro-CHOEP and after HDT and transplantation, the OS (defined as the time between the date of enrollment and the date of death from any cause in the ITT population enrolled in the study). Safety secondary endpoints included toxicities during the induction and in all treatments, recorded and classified according to the definitions of NCI CTCAE, v4.0. The treatment-related mortality is defined as any death that was not attributable to the lymphoma.

### Statistical analyses

#### Phase Ib

The continual reassessment method (CRM) for dose-finding phase I study [17, 18] was used to assess the MTD of romidepsin when administered in combination with CHOEP in the treatment of patients with PTCL, candidate to HDT. The MTD was defined as the dose of romidepsin that achieved a DLT in 33% of patients. Four dose levels were tested, namely 8, 10, 12, and 14 mg/ms. On the basis of the available evidence at the time of study initiation, the first three included patients were administered the third dose level (12 mg/ms). After the enrollment of the first three patients, accrual continues, with grouped inclusions of three patients per dose level. Then, based on observed responses (DLT or not), DLT probabilities of all dose levels were updated using Bayes theorem. The dose level associated with an updated DLT probability close to 33% was recommended to be administered to the next patient cohort. All this process was re-run until the fixed sample size (*N* = 24) was reached, or until the upper and lower bounds of the 95% credibility interval of the estimated probability of DLT at the final recommended dose were between 16% and 60% (precision rule), or in case of fulfilled stopping criteria measuring futility of trial continuation [19]. The CRM design was implemented using the “bcrm” package of R 3.1.0 and later versions (R Development Core Team, Vienna, Austria).

#### Phase II

The sample size of the phase II part of the trial was calculated using the PFS as the primary endpoint, according to the two-stage design proposed by Case and Morgan [20], without interim pause in the enrollment. According to available evidence, the 18 months PFS of newly diagnosed PTCL patients treated with anthracycline-based therapy, followed by transplantation, was around 55% (null hypothesis, H0). With our experimental strategy, we hypothesized to achieve an overall 18 months PFS of 70% (alternative hypothesis, H1).

To demonstrate an absolute improvement from 55% (literature data) to 70% of the 18 months PFS, with an alpha error of 0.05 (one tail), a beta error of 0.10, and assuming 3 years of constant accrual and at least 18 months of follow-up after the enrollment of the last patient, the required total sample size calculated to minimize the expected total study length was 110 (sample size calculated with the Sample Size Tables for Clinical Studies, 3rd edition, by Machin et al. [21]). With this design, the interim analysis was planned when the first 75 patients have been enrolled. At this time, the Kaplan-Meier 18 months PFS would be estimated, with its standard error, to calculate the Z interim test; the study would be completed if the threshold of the Z interim test for efficacy was at least 0.650. If this case, 35 further patients would be enrolled to reach the planned total sample size of 110. To reject the null hypothesis the threshold of the Z final statistic would be greater than 1.522.

An exploratory analysis of prognostic factors for PFS and OS was also performed using multivariable Cox models, evaluating histological subgroups (PTCL-NOS, ALK-negative, AITL/THF), age at enrollment, bone marrow involvement, abnormal LDH, Ann Arbor stage, ECOG PS, extranodal involvement.

Statistical analyses were performed using Stata, version 14 (StataCorp).

## Results

### Phase Ib part

From 2014 to 2017, 21 patients were enrolled in phase Ib part. Median age was 57 years (IQR 53;61); 18 (86%) had an Ann Arbor stage III–IV and 8 (38%) an International Prognostic Index (IPI) > 2. The first triplet was treated with Ro at 12 mg/ms, and no DLTs were observed; the subsequent cohorts were treated with Ro at 14 mg/ms. Nine DLTs were reported in seven patients: eight events of grade 3 (three cases of mucositis, one of maculopapular rash, fatigue, fever, respiratory failure, bowel typhlitis) and one event of g4 neutropenic fever. According to the continual reassessment method, the observed DLTs prompted to define 14 mg/ms the recommended dose of romidepsin, with an estimated DLT probability of 34.8% (95% Credibility Interval 16.9–57.0). At the end of induction, ORR was 16/21 (76%), with CR 71%.

### Phase II part

The interim analysis according to the Case and Morgan design was performed upon reaching the 79th enrolled patient. In the period that elapsed for the preparation of the interim report and the decision to stop enrollment, a further 7 patients were enrolled. Thus, a total of 86 patients were evaluable at MTD: 68 patients in phase II and 18 previously treated with romidepsin at 14 mg/ms during the phase Ib part of the study.

In accordance with the statistical design, the interim analysis was performed upon reaching the 75th patient enrolled, and all 86 patients receiving romidepsin at 14 mg/ms were considered for the interim analysis. Clinical characteristics are reported in Table 1. Median age was 55 years (IQR 49;60); 78 (91%) had Ann Arbor stage III-IV and 12 (14%) Eastern Cooperative Oncology Group performance status >1; IPI score was reported >2 in 29 (34%) and Prognostic Index for T-cell lymphoma (PIT) score >1 in 34 (43%) patients; BM involvement was present in 31 (36%) patients. According to central pathological review, histological subgroups were: 34 (40%) PTCL-NOS, 21 (24%) ALK-negative, and 31 (36%) AITL/THF.

**Table 1:** Patients characteristics.

Factor	Level	Value
*N*		86
Age at enrollement, median (IQR)		55 (49, 60)
Gender	Female	28 (33%)
	Male	58 (67%)
Histology by Investigators	PTCL-NOS or ALCL ALK neg	66 (77%)
	AITL	20 (23%)
Histological review	ALCL ALK neg	21 (24%)
	PTCL-NOS	34 (40%)
	T HELPER FOLLICULAR/AITL	31 (36%)
IPI Score	0	5 (6%)
	1	30 (35%)
	2	20 (23%)
	>=3	31 (36%)
PIT Score	0	19 (24%)
	1	26 (33%)
	2	20 (25%)
	>= 3	14 (18%)
Bone Marrow involved	0	54 (64%)
	1	31 (36%)
LDH Abnormal	No	43 (50%)
	Yes	43 (50%)
Ann Arbor Stage	II	8 (9%)
	III	24 (28%)
	IV	54 (63%)
ECOG PS	0	51 (59%)
	1	23 (27%)
	2	11 (13%)
	3	1 (1%)
N extrasites	0	37 (43%)
	1	28 (33%)
	2	13 (15%)
	>= 3	8 (9%)

*N* number, *PTCL-NOS* peripheral T-cell lymphoma not otherwise specified, *ALCL ALK neg* anaplastic large cell lymphoma anaplastic lymphoma kinase negative, *AITL* angioimmunoblastic T-cell lymphoma, *IPI score* International Prognostic Index score, *PIT score* Prognostic Index for T-cell lymphoma score, *LDH* lactate dehydrogenase, *ECOG PS* Eastern Cooperative Oncology Group Performance Status.

**Fig. 1:**
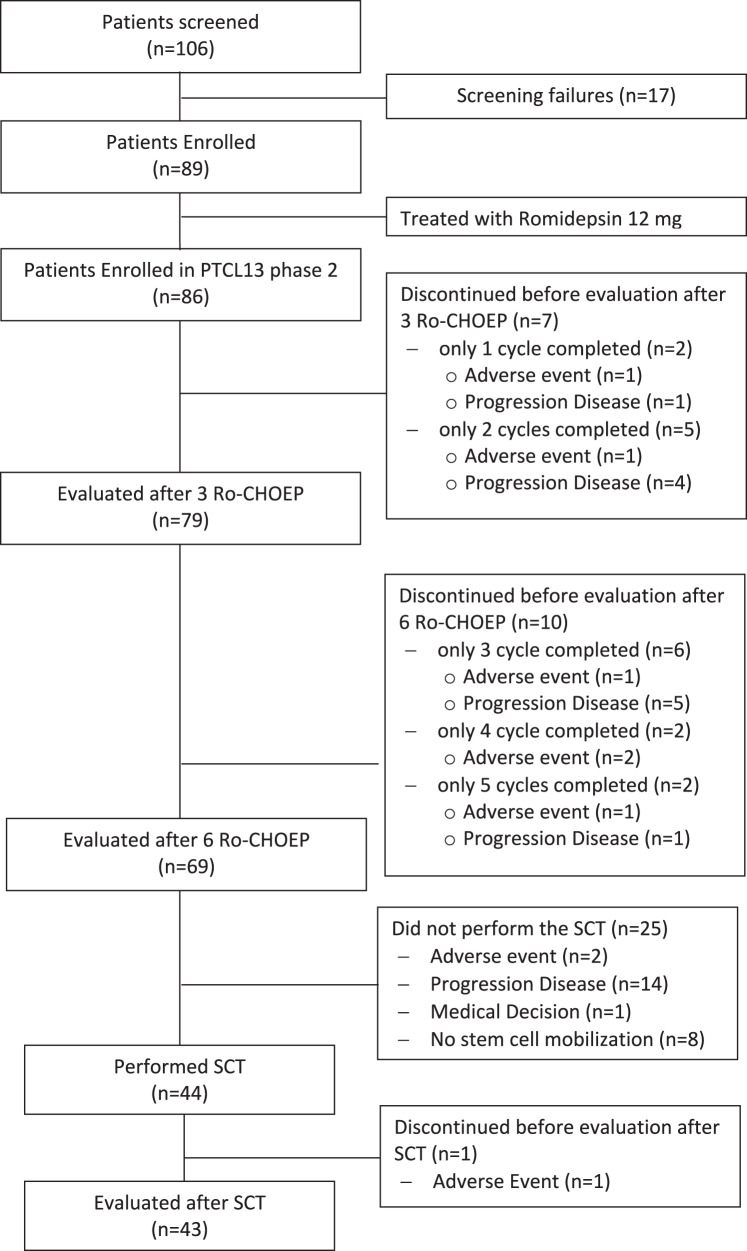
Flowchart. PTCL: peripheral T-cell lymphoma; Ro-CHOEP: Romidepsin, cyclophosphamide, doxorubicin, vincristine, etoposide, prednisone; SCT: stem cell transplantation.

After 3 Ro-CHOEP ORR was 87.2% (75 patients), with 32.6% (28) CR. After 6 Ro-CHOEP, ORR was 70.9% (61), with 61.6% (53) CR. (Fig. 1).

Among the 86 patients evaluable for response at the end of treatment after SCT, the final ORR was 57% (49 patients), with 56% (48) CR.

Forty of 53 patients in CR at end of induction underwent SCT (39 auto-SCT, 1 allo-SCT due to mobilization failure); at restaging after SCT 35 (87.5%) were in CR. Four of eight patients in PR underwent SCT: two received allo-SCT and two auto-SCT (one medical decision, one lack of donor). One patient experienced an adverse event after allo-SCT (septic shock) whereas at restaging, one patient converted to CR, one patient maintained PR, one patient had PD.

Overall 42 of 86 were not transplanted for different reasons: 25 progressive disease, 8 poor mobilizers, 8 adverse events (3 sepsis, 1 cytomegalovirus infection, 2 cardiological events, 1 myelodysplastic syndrome, and 1 withdrawal from the study due to an infusion reaction to romidepsin), 1 medical decision (patient too frail to underwent SCT).

The interim analysis performed on the first 79 patients enrolled (August, 2020) observed a PFS at 18 months of 53%, resulting into a Z interim test = -0.357, clearly lower than the minimum value required for trial continuation (Z = 0.650), therefore the enrollment was stopped for inefficacy.

**Fig. 2:**
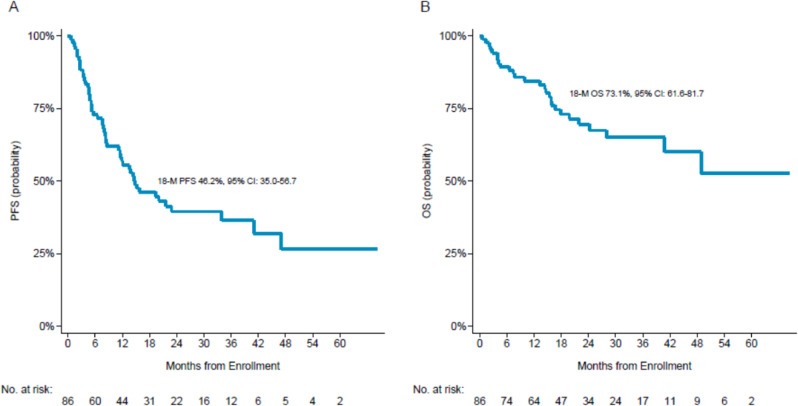
Progression-free survival and Overall Survival at 18-months. **A** Progression free survival (PFS); **B** Overall survival (OS).

Considering the all cohort of 86 patients, at a median follow-up of 28 months, the 18-month PFS was 46.2% (95% CI: 35.0–56.7) and the OS was 73.1% (95% CI: 61.6–81.7), Fig. 2. The 18-month PFS for PTCL-NOS versus ALK-negative versus AITL/THF was 35.1% (95% CI: 19.7–50.9) vs. 51.4% (95% CI: 28.4–70.4) vs. 56.4% (95% CI: 36.0–72.5), log-rank test p 0.149; the 18-months OS for PTCL-NOS vs. ALK-negative vs. AITL/THF was 67.6% (95% CI: 47.7–81.3) vs. 70.2% (95% CI: 45.1–85.4) vs. 81.5% (95% CI: 60.9–91.9), log-rank test *p* = 0.685. These results were confirmed in multivariable analysis (Supplementary Table 1). Among the prognostic factors evaluated, patients with Ann Arbor stage IV showed a significantly higher risk of progression compared to stages II-III (HR = 2.84, 95%CI, 1.17–6.90, *p* = 0.021).

The most frequent toxicities during Ro-CHOEP treatment were hematological, with grade 3–4 neutropenia and thrombocytopenia in 33% and 34% of all the 459 cycles, respectively; severe febrile neutropenia was reported in only 5% of Ro-CHOEP courses. Severe non-hematological toxicities were observed in 30 (34.9%) of patients: cardiological in 5 patients (6%), gastrointestinal in 9 (10%), infections in 10 (12%), others in 11 (13%); (Tables 2, 3; Supplementary Table 1).

**Table 2:** Hematological adverse events according to CTCAE 4.0 by Ro-CHOEP cycles (number of cycles = 459).

	Grade 1–2	Grade 3	Grade 4
Any type	41 (8.93)	63 (13.73)	156 (33.99)
Anemia	61 (13.29)	50 (10.89)	6 (1.31)
Leucopenia	6 (1.31)	19 (4.14)	43 (9.37)
Neutropenia	11 (2.4)	43 (9.37)	110 (23.97)
Thrombocytopenia	26 (5.66)	52 (11.33)	103 (22.44)
Febrile Neutropenia	7 (1.53)	10 (2.18)	11 (2.4)

Data are *n* (%). *CTCAE* Common Terminology Criteria for Adverse Events.

**Table 3:** Max non-hematological adverse events according to CTCAE 4.0 by patient during Ro-CHOEP cycles (number of patients: 86).

	Grade 1–2	Grade 3	Grade 4
Any Type	44 (51.16)	22 (25.58)	8 (9.3)
Cardiac disorders	7 (8.14)	4 (4.65)	1 (1.16)
Eye disorders	1 (1.16)	1 (1.16)	0 (0)
Gastrointestinal disorders	34 (39.53)	9 (10.47)	0 (0)
General disorders and administration site conditions	20 (23.26)	3 (3.49)	1 (1.16)
Hepatobiliary disorders	1 (1.16)	0 (0)	0 (0)
Immune system disorders	1 (1.16)	1 (1.16)	0 (0)
Infections and infest	15 (17.44)	8 (9.3)	2 (2.33)
Injury, poisoning, and procedural complications	2 (2.33)	0 (0)	0 (0)
Investigations	6 (6.98)	0 (0)	0 (0)
Metabolism and nutrition disorders	9 (10.47)	2 (2.33)	1 (1.16)
Musculoskeletal and connective tissue disorders	2 (2.33)	0 (0)	0 (0)
Neoplasms benign, malignant, and unspecified	1 (1.16)	0 (0)	0 (0)
Nervous system disorders	7 (8.14)	2 (2.33)	0 (0)
Renal and urinary disorders	1 (1.16)	0 (0)	0 (0)
Reproductive system and breast disorders	1 (1.16)	0 (0)	0 (0)
Respiratory, thoracic, and mediastinal disorders	8 (9.3)	3 (3.49)	0 (0)
Skin and subcutaneous tissue disorders	4 (4.65)	2 (2.33)	1 (1.16)
Vascular disorders	7 (8.14)	0 (0)	0 (0)
Other	16 (18.6)	8 (9.3)	3 (3.49)

Data are *n* (%). CTCAE: Common Terminology Criteria for Adverse Events.

A median of 5.0 × 10^6^ (IQR 3.4–6.9) peripheral blood CD34 + cells/kg was collected during the harvest. At least 90% of the planned dose of doxorubicine, cyclophosphamide, etoposide, and vincristine was administered in 94%, 93%, 89%, and 98% of cycles, respectively. Median RTDI of romidepsin was 87.7% (IQR: 69.5–94.6%; range: 8.2–100.1%). Median interval time between Ro-CHOEP was 21 days (IQR: 21–22; range: 19–69). Granulocyte-colony stimulating factors was administered in 424/459 (92.4%) cycles of therapy.

Twenty-seven patients died: 22 for lymphoma progression, one for transplant-related mortality after allo-SCT, 3 for complications of other salvage therapy, and one for fungal pneumonia.

## Discussion

The treatment of advanced-stage PTCLs is still an unmet clinical need with an overall cure rate of ~45%. The main problem relies on the fact that ~30% of patients are primary refractory or have an early disease progression. In order to improve these disappointing results, we designed a trial to test the combination of romidepsin with chemotherapy before SCT. Based on the results of the Nordic trial, we decided to adopt the regimen CHOEP, with the addition of etoposide to standard CHOP, as induction chemotherapy backbone for our study, devoted to young patients. The rationale was to combine the supposed more effective chemotherapy with an HDAC inhibitor to increase the remission rate before the transplant phase. An increase in remissions would have led to a higher number of patients receiving transplant consolidation, to hopefully improve the final cure rate. Considering the dismal outcome of PTCL, the trial explored also the possibility of upfront allografting for patients only in PR after induction. We and others showed that allogeneic SCT is an effective salvage treatment for chemosensitive relapsed PTCLs [16, 22–24]. For the above-mentioned reasons, patients in less than PR were considered as failures and underwent salvage treatments according to institutional policies.

With a Bayesian statistical design during the phase Ib part, we defined romidepsin 14 mg/ms as the MTD. To our knowledge, this is the only trial defining the MTD of romidepsin with CHOEP in a program including a final transplantation. In the French trial, the recommended dose was 12 mg/ms in combination with CHOP, and the same dose was applied to the randomized study, but their study did not include a transplant phase [13, 14].

Unfortunately, the PTCL13 study did not meet the primary endpoint, with a PFS at 18 months of 48% (95% CI: 0.36–0.58) during the planned interim analysis, therefore the trial was stopped due to inefficacy of the experimental combination. Our results are superimposable to those reported by Bachy in the randomized trial, with PFS rates at 1 year and 2 years of 49.8%, and 43.2%, respectively, in the Ro-CHOP arm. The French trial results were obtained without transplantation, thus raising some doubts on its role in the upfront treatment of PTCLs.

In our study, the outcome of AITL/THF was similar to PTCL-NOS. This finding, however, must be interpreted with caution because the subgroups are very small and the trial was not powered to see such differences.

In the PTCL13 study, the combination was well tolerated, with no unexpected toxicities, and the most frequent adverse events were hematological, in particular grade ≥3 neutropenia (33% of all courses) and thrombocytopenia (34% of all courses). The use of romidepsin had not a detrimental effect on stem cell mobilization. The most frequent cause of treatment discontinuation was disease progression. In agreement with Bachy et al., we can conclude that romidepsin did not ameliorate the prognosis of PTCL patients, when added to chemotherapy, not even in our study that included a final transplant phase. PFS results are superimposable to those obtained with CHOEP alone and HDT by D’Amore et al.

One more issue is the role of frontline allogeneic SCT that is a matter of debate since a decade. Recently, a phase III randomized trial on 104 untreated PTCLs patients reported, at a median follow-up of 42 months, a 3-year EFS after allo-SCT of 43%, compared with 38% after auto-SCT and a 3-year OS of 57% versus 70%, respectively. No significant differences were demonstrated between the two arms because the postulated graft-versus-lymphoma effect was counterbalanced by the higher transplant-related mortality in the allo-SCT group. Although the allo-SCT arm had no relapses, supporting a graft versus lymphoma effect [25].

In our study, of the eight patients in PR after six Ro-CHOEP induction, only three underwent allo-SCT, and one died of TRM, supporting the notion that there is no role for frontline allo-SCT.

Several biological agents have been tested in combination with chemotherapy, but only brentuximab-vedotin, a monomethyl-auristatin-E-antibody conjugate directed against CD30, used in combination with chemotherapy (A-CHP), showed in a phase III trial an advantage compared to CHOP in the first line treatment of CD30 + PTCLs, with no unexpected toxicities; however, 70% of the enrolled patients were anaplastic T-cell lymphoma, and more than 20% of patients received consolidation with SCT [26]. Thus the results cannot be compared with the present study.

In conclusion, we think that only a better knowledge of disease biology will allow the design of trials with novel drug combinations specific for genetic subgroups of PTCLs. The recent results of romidepsin plus 5-azacytidine in relapsed/refractory AITL/THF [27], are in line with this approach and will be the basis for future studies.

## Supplementary information

Supplementary materials

study protocol

## Data Availability

The dataset analyzed during the current study is available from the corresponding author on reasonable request.
