# Supplementary material for: Romidepsin-CHOEP followed by high-dose chemotherapy and stem-cell transplantation in untreated Peripheral T-Cell Lymphoma: results of the PTCL13 phase Ib/II study
Source: Leukemia. 2023 Jan 18;37(2):433–40. doi: 10.1038/s41375-022-01780-1 (PMC9898022; doi:10.1038/s41375-022-01780-1)
Supplement: Supplementary file 1 — Supplementary materials [file 41375_2022_1780_MOESM1_ESM.docx]

**Supplementary Appendix**

**Table of contents:**

1. Title
2. Investigators
3. Patient selection criteria
   1. Inclusion criteria
   2. Exclusion criteria
4. Study design
   1. Figure 1s
   2. Concomitant Supportive Medications
5. Table 1s: Max haematological adverse events according to CTCAE 4.0 by patient during Ro-CHOEP cycles
6. Table 2s: Multivariable analysis. Cox proportional-hazards model.

**Title.**

**Romidepsin plus CHOEP followed by high-dose chemotherapy and stem-cell transplantation in young patients with previously untreated Peripheral T-Cell Lymphoma: results of the PTCL13 phase Ib/II study.**

**Investigators.**

List of centers and investigators of Fondazione Italiana Linfomi (FIL).

Division of Hematology and Stem Cell Transplantation, Fondazione IRCCS Istituto Nazionale dei Tumori, Milano, Italy: P. Corradini, A. Chiappella, A. Dodero, G. Perrone, L. Farina, M. Pennisi, A. Guidetti, P. Matteucci, L. Devizzi, C. Rusconi.

Hematology Division, ASST Spedali Civili di Brescia, Brescia, Italy: A. Re, A. Tucci, G. Rossi

Division of Hematology, Azienda Ospedaliera e Universitaria Città della Salute e della Scienza, Torino, Italy: L. Orsucci, B. Botto, C. Boccomini, U. Vitolo, R. Freilone

Hematology Unit, Ospedale Oncologico Armando Businco, Cagliari, Italy: G. Cabras, S.V. Usai

Division of Hematology, Azienda Ospedaliera S. Croce e Carle, Cuneo, Italy: C. Castellino, M. Massaia

Institute of Hematology "Seràgnoli", University of Bologna, Bologna, Italy: V. Stefoni, P.L. Zinzani

Hematology-Oncology & Stem Cell Transplantation Unit, Istituto Nazionale Tumori, Fondazione G. Pascale, IRCCS, Napoli, Italy: A. Pinto

Division of Hematology, Azienda Ospedaliera Santi Antonio e Biagio e Cesare Arrigo, Alessandria, Italy: M. Zanni, F. Salvi, M. Ladetto

Onco-hematology and Stem Cell Transplantation and Cellular Therapies, Centro di Riferimento Oncologico di Aviano (CRO) IRCCS, Aviano, Italy: R. Ciancia, M. Spina

Unit of Hematology and Cellular therapies, IRCCS Ospedale Policlinico San Martino, Genova, Italy: C. Ghiggi, E. Angelucci

Division of Hematology, Fondazione IRCCS Cà Granda, OM Policlinico, Milano, Italy: F.G. Rossi, L. BaldiniHematology Unit, Ospedale Guglielmo da Saliceto, Piacenza, Italy: A. Arcari, D. Vallisa

Hematology, Azienda USL-IRCCS di Reggio Emilia, Reggio Emilia, Italy: F. Ilariucci, S. Luminari, F. Merli

Division of Hematology, ASST Grande Ospedale Metropolitano Niguarda, Milano, Italy: V.R. Zilioli, R. Cairoli

Hematology, Azienda Ospedaliera di Perugia, Perugia, Italy: L. Flenghi

Hematology, Ospedale degli Infermi, Rimini, Italy: A.L. Molinari, M. Celli

Clinic of Hematology, Presidio Ospedaliero Universitario "Santa Maria della Misericordia” di Udine, ASUFC, Udine, Italy: S. Volpetti, F. Patriarca

Hematology and Stem Cell Transplantation, Azienda Ospedaliera Universitaria di Verona, Verona, Italy: F. Benedetti

Hematology, IRCCS Ospedale Policlinico San Martino, Genova, Italy: F. Ballerini, R.M. Lemoli

Hematology unit, IRCCS Istituto Romagnolo per lo Studio dei Tumori (IRST) "Dino Amadori", Meldola, Italy: G. Musuraca

Division of Hematology, Ospedale Maggiore Della Carità, Novara, Italy: R. Bruna, G. Gaidano

Division of Onco-Hematology, Azienda Villa Sofia Cervello, Palermo, Italy: C. Patti

Hematology and CTMO, Azienda Ospedaliera-Universitaria di Parma, Parma, Italy: F. Re, F. Leonardi

Department of Molecular Medicine,University of Pavia & Division of Hematology, Fondazione IRCCS Policlinico San Matteo, Pavia, Italy: L. Arcaini

Department of Oncology and Hematology, Humanitas Cancer Center, IRCCS Humanitas Research Hospital, Rozzano, Italy: M. Magagnoli, A. Santoro

Division of Hematology, Department of Molecular Biotechnologies and Health Sciences, University of Torino/AOU "Città della Salute e della Scienza di Torino: F. Cavallo, D. Caracciolo, B. Bruno

Laboratory of Hematology, Division of Hematology and Stem Cell Transplantation, Fondazione IRCCS Istituto Nazionale dei Tumori, Milano, Italy: C. Carniti, E. Fardella

Division of Haematopathology, European Institute of Oncology IRCCS, Milano, Italy: S.A. Pileri, V. Tabanelli

Unit of Clinical Epidemiology, Azienda Ospedaliera e Universitaria Città della Salute e della Scienza and CPO Piemonte, Torino, Italy: G.Ciccone, A. Evangelista.

Trial Office FIL staff: Sonia Perticone, Claudia Peracchio, Emanuela Anna Pesce, Elena Borgo, Anna Fedina.

**PATIENT SELECTION CRITERIA**

Newly diagnosed patients with Peripheral T-cell lymphomas including: Peripheral T-cell

lymphomas not otherwise specified (PTCL-NOS), Angioimmunoblastic T-cell lymphoma (AITL),

ALK negative Anaplastic large-cell lymphoma (ALCL).

**Inclusion criteria**

1. age ≥18 e ≤ 65 years

2. Peripheral T-cell lymphomas at diagnosis including: PTCL-NOS, AITL, ALK negative

ALCL

3. Stage II-IV

4. Written informed consent

5. No prior treatment for lymphoma

6. No Central Nervous System (CNS) disease (meningeal and/or brain involvement by

lymphoma)

7. HIV negativity

8. Absence of active hepatitis C virus (HCV) infection

9. HBV negativity or patients with HBcAb +, HBsAg -, HBs Ab+/- with HBV-DNA negativity

(in these patients Lamivudine prophylaxis is mandatory)

10. Levels of serum bilirubin, alkaline phosphatase and transaminases < 2 the upper normal limit, if not disease related

11. No psychiatric illness that precludes understanding concepts of the trial or signing informed consent

12. Ejection fraction > 50% and no myocardial stroke in the last year nor QT prolongation (QTc interval < 480 msec using the Fridericia formula)

13. Clearance of creatinine > 60 ml/min if not disease related

14. Spirometry Diffusion Capacity (DLCO) > 50%

15. Absence of active, uncontrolled infection

16. For males and females of child-bearing potential, agreement upon the use of effective

contraceptive methods prior to study entry, for the duration of study participation and in the

following 90 days after discontinuation of study treatment

17. Availability of histological material for central review and pathobiological studies.

**Exclusion criteria**

1. age <18 e > 65 years

2. Hystology other than: PTCL-NOS, AITL, ALKnegativeALCL

3. Stage I

4. Prior treatment for lymphoma

5. Positive serologic markers for human immunodeficiency virus (HIV)

6. Active hepatitis B virus (HBV) infection

7. Active hepatitis C virus (HCV) infection

8. Levels of serum bilirubin, alkaline phosphatase and transaminases > 2 the upper normal

limit, if not disease related

9. Ejection fraction < 50% and myocardial stroke in the last year or QT prolongation (QTc

interval > 480 msec using the Fridericia formula)

10. Clearance of creatinine < 60 ml/min if not disease related

11. Spirometry Diffusion Capacity (DLCO) < 50%

12. Pregnancy or lactation

13. Patient not agreeing to take adequate contraceptive measures during the study

14. Psychiatric disease that precludes understanding concepts of the trial or signing informed consent

15. Any active, uncontrolled infection

16. Prior history of malignancies other than PTCLs in the last five years (except for basal cell or squamous cell carcinoma of the skin or carcinoma in situ of the cervix or breast).

**Study design**

**Figure 1s. Study design.**

In details, patients received Ro-CHOEP every 21 days (750 mg/ms intravenous cyclophosphamide, 50 mg/ms intravenous doxorubicin, etoposide 100 mg/ms intravenous on days 1 through 3, and 1.4 mg/ms intravenous vincristine [capped at 2.0 mg] on day 1, and 40 mg/ms oral prednisone or equivalent intravenous on days 1–5). After MTD definition, romidepsin 14 mg/ms was administered by a 4-hours infusion, on day 1 and on day 8; for logistic reasons, the infusion of romidepsin was allowed also on day 0.

**Concomitant Supportive Medications**

All patients received primary prophylaxis for neutropenia with granulocyte colony-stimulating factors, and for Pneumocystis jirovecii infection with co-trimoxazole or a pentamidine aerosol. Occult carriers of hepatitis B virus were given lamivudine. All supportive therapies, in particular those concerning anti-nausea treatment, CMV prophylaxis, anti-fungal prophylaxis or the treatment of infections, considered a standard practice for the induction and for the consolidation with auto or allo-SCT were permitted.

**Table 1s: Max haematological adverse events according to CTCAE 4.0 by patient during Ro-CHOEP cycles (N=86)**

|  | Grade 1-2 | Grade 3 | Grade 4 |
| --- | --- | --- | --- |
|  |  |  |  |
| Any Type | 7 (8.14) | 8 (9.3) | 56 (65.12) |
| Anemia | 17 (19.77) | 27 (31.4) | 6 (6.98) |
| Leucopenia | 1 (1.16) | 2 (2.33) | 23 (26.74) |
| Neutropenia | 2 (2.33) | 9 (10.47) | 46 (53.49) |
| Thrombocytopenia | 6 (6.98) | 14 (16.28) | 40 (46.51) |
| Febrile Neutropenia | 4 (4.65) | 10 (11.63) | 12 (13.95) |

Data are n (%). CTCAE=Common Terminology Criteria for Adverse Events.

**Table 2s: Multivariable analysis. Cox proportional-hazards model.**

|  | Progression-Free Survival | | | Overall Survival | | |
| --- | --- | --- | --- | --- | --- | --- |
| Fistological subgroup | HR | 95%CI | p | HR | 95%CI | p |
| PTCL-NOS (ref) | 1 | - | - | 1 | - | - |
| ALK-negative | 0.95 | 0.45,2.03 | 0.900 | 1.04 | 0.38,2.88 | 0.936 |
| AITL/THF | 0.55 | 0.27,1.09 | 0.085 | 0.85 | 0.32,2.23 | 0.739 |
|  |  |  |  |  |  |  |
| Age, per 1-y increase | 1.03 | 1.00,1.07 | 0.085 | 1.03 | 0.98,1.09 | 0.201 |
| Bone Marrow involved | 0.74 | 0.34,1.59 | 0.439 | 0.44 | 0.16,1.16 | 0.096 |
| Abnormal LDH | 0.70 | 0.37,1.36 | 0.296 | 1.02 | 0.41,2.57 | 0.963 |
| Stage IV vs II-III | 2.84 | 1.17,6.90 | 0.021 | 2.57 | 0.83,7.97 | 0.103 |
| ECOG PS≥2 | 2.21 | 0.91,5.36 | 0.079 | 2.32 | 0.75,7.12 | 0.143 |
| N. Extranodal≥1 | 0.65 | 0.33,1.28 | 0.211 | 1.08 | 0.42,2.76 | 0.874 |
