# Supplementary material for: Romidepsin-CHOEP followed by high-dose chemotherapy and stem-cell transplantation in untreated Peripheral T-Cell Lymphoma: results of the PTCL13 phase Ib/II study
Source: Leukemia. 2023 Jan 18;37(2):433–40. doi: 10.1038/s41375-022-01780-1 (PMC9898022; doi:10.1038/s41375-022-01780-1)
Supplement: Supplementary file 2 — study protocol [file 41375_2022_1780_MOESM2_ESM.pdf]

STUDY PROTOCOL

ROMIDEPSIN IN COMBINATION WITH CHOEP AS FIRST LINE TREATMENT BEFORE HEMATOPOIETIC STEM CELL TRANSPLANTATION IN YOUNG PATIENTS WITH NODAL PERIPHERAL T-CELL LYMPHOMAS: A PHASE I-II STUDY.

STUDY DRUG Romidepsin
Study ID Phase I-II FIL_PTCL13
EUDRACT Number 2013-005179-41
Version 2.0 July 22th, 2015

STUDY CONTACT INFORMATION

INVESTIGATOR AND SPONSOR

Fondazione Italiana Linfomi Onlus (FIL)

Study FIL Office: c/o Azienda Ospedaliera SS. Antonio e Biagio e Cesare Arrigo

Address: via Venezia 16, 15121 Alessandria, Italy

Phone no.: +39-0131-206071-206288

Fax no.: +39-0131-263455

Email: segreteria@filinf.it

PRINCIPAL INVESTIGATOR

Prof. Paolo Corradini

Address: Department of Hematology - Fondazione IRCCS Istituto Nazionale dei Tumori, via Venezian 1, 20233 Milano, University of Milan, Italy

Phone n. +39(0)2 2390 2950

Fax n. +39(0)2 2390 2908

E-mail: paolo.corradini@unimi.it

WRITING COMMITTEE AND SCIENTIFIC SUPPORT

- Paolo Corradini, Fondazione IRCCS Istituto Nazionale dei Tumori, Milano, Italy: design of the study and protocol writing
- Annalisa Chiappella, AO Città della Salute e della Scienza, Torino, Italy: design of the study and protocol writing
- Giulia Perrone, Fondazione IRCCS Istituto Nazionale dei Tumori, Milano, Italy: design of the study and protocol writing
- Umberto Vitolo, AO Città della Salute e della Scienza, Torino, Italy: revision of the study and protocol writing
- Pierluigi Zinzani, Istituto Seragnoli, Università degli Studi, Bologna, Italy: revision of the study and protocol writing
- Giuseppe Rossi, Spedali Civili, Brescia, Italy: revision of the study and protocol writing
- Francesco Zaja, DIRM, AOU Santa Maria della Misericordia, Udine, Italy: revision of the study and protocol writing
- Giovannino Ciccone, AO Città della Salute e della Scienza e CPO Piemonte, Torino, Italy: statistical design

BIOMETRY

Responsible: Giovannino Ciccone, MD

Address: SSCVD Epidemiologia Clinica e Valutativa – AO Città della Salute e della Scienza di Torino e CPO Piemonte, corso Bramante 88, Torino, Italy

Phone no.: +39-011-6336857

Fax no.: +39-011-6334571

PHARMACOVIGILANCE

Responsible: Alessandro Levis, MD

Address: Study FIL Office c/o Azienda Ospedaliera S.S. Antonio e Biagio e Cesare Arrigo - Alessandria

Phone no.: +39-0131-206071-206066

Fax no.: +39-0131-261029

REFERENCE LABORATORY FOR MOLECULAR BIOLOGY

Responsible: Dr. Cristiana Carniti

Address: Laboratorio di Ematologia – Trapianto di Midollo Osseo Allogenico

Fondazione IRCCS, Istituto Nazionale dei Tumori

via Venezian, 1 20133 Milano, Italy

REFERENCE HEMO-PATHOLOGY LABORATORY

Responsible: Prof. Stefano Pileri

Address: Haematopathology Unit

European Institute of Oncology

Via Ripamonti 435 – 20141 Milano (Italy)

stefano.pileri@ieo.it

INVESTIGATOR AGREEMENT

I have read this protocol and agree that it contains all necessary details for carrying out this study. I will conduct the study as outlined herein and will complete the study within the time designated.

I will provide copies of the protocol and all pertinent information to all individuals responsible to me who assist in the conduct of this study. I will discuss this material with them to ensure that they are fully informed regarding the study drug and the conduct of the study.

____

Investigator’s Signature Date

____

Name of Investigator (Typed or Printed)

____

____

____

Institution, Address*

____

Phone Number*

____

Investigator-Sponsor Signature* Date
(where required)

____

Name of Coordinating Investigator (Typed or Printed)

____

Institution

____

* If the address or phone number of the investigator changes during the course of the study, written notification will be provided by the investigator to the sponsor and will not require protocol amendment(s).

INDEX

1 SYNOPSIS 8

2 FLOW CHART 17

3 BACKGROUND AND INTRODUCTION 18

3.1 Study background 18

3.2 Rational of the study 20

3.3 Romidepsin 21

3.4 Romidepsin Safety Profile 21

3.4.1 Identified and Potential Risks of Romidepsin 21

3.4.2 Special Risk Considerations for Romidepsin 25

3.4.3 Special risk considerations for combination Romidepsin CHOP 26

4 PATIENT SELECTION CRITERIA 27

4.1 Inclusion criteria 27

4.2 Exclusion criteria 27

5 PHASE I PART OF THE STUDY 28

5.1 Objectives of the study 28

5.2 End-points 28

5.3 Statistical design 28

5.3.1 Study population 29

5.3.2 Study design and treatment 29

6 PHASE II PART OF THE STUDY 29

6.1 Objectives of the study 29

6.2 Endpoints 30

6.3 Statistical design 30

6.3.1 Study population 31

6.3.2 Duration of the study 31

7 STUDY TREATMENT 31

7.1 Registration of the patient and Romidepsin dose-allocation 36

8 PATHOLOGICAL REVIEW AND BIOLOGIC STUDIES 36

8.1 Pathological review 36

8.2 Biological studies 36
8.3 Blood for germline DNA 37
8.4 Operative considerations for sample shipment for the biological studies 37
8.5 Ethical Aspects of Biological studies 37
9 STUDY TREATMENT AND CONCOMITANT TREATMENT 38
9.1 Dose-adjustment for Romidepsin 38
9.2 Dose- adjustment for CHOEP 38
9.3 Dose -adjustment for DHAP 38
9.4 Recommended concomitant treatments 38
9.5 Permitted concomitant therapy 39
9.6 Prohibited concomitant therapy 39
9.7 Drugs affecting Qtc 39
9.8 Inhibitor or inducer of Cytochrome P450 3A4 Enzyme 39
9.9 Inhibitor or drug transport systems 39
10 CLINICAL EVALUATION, LABORATORY TESTS AND FOLLOW-UP 40
10.1 Staging evaluation, baseline 40
10.2 Evaluation at each Ro-CHOEP courses 40
10.3 Intermediate response evaluation 40
10.4 Post-Induction evaluation 40
10.5 Post –SCT evaluation 41
10.6 Follow-up 41
11 FORMS AND PROCEDURES FOR COLLECTING DATA AND DATA MANAGING 41
12 ADVERSE EVENTS, SERIOUS ADVERSE EVENTS 42
12.1 Adverse Event 42
12.2 Serious Adverse Event 42
12.3 Unlisted (Unexpected) Adverse Event 43
12.4 Associated with the Use of the Drug 43
12.5 Product Quality Complaint 43

12.6 **Attribution Definitions** **43**
12.6.1 Intensity (Severity) Reporting and Attribution 43

12.7 **Reporting Procedures** **44**

13 ETHICAL CONSIDERATIONS **47**

13.1 **Patient protection** **47**

14 SUBJECT IDENTIFICATION – PERSONAL DATA PROTECTION **47**

14.1 **Informed consent** **48**

15 CONFLICT OF INTEREST **48**

16 DATA OWNERSHIP **48**

17 PUBLICATION POLICY **48**

18 STUDY INSURANCE **49**

19 REFERENCES **49**

20 APPENDIXES **52**

1 SYNOPSIS

**PROTOCOL TITLE**	**Romidepsin in combination with CHOEP as first line treatment before hematopoietic stem cell transplantation in young patients with nodal peripheral T-cell lymphomas: a phase I-II study.**
**PROTOCOL VERSION**	**n=2 July 22th, 2015**
**SPONSOR**	Fondazione Italiana Linfomi (FIL)
**PROTOCOL PHASE:**	This is a multicenter study that includes two phases:<br>1. A phase I study to define the maximum tolerated dose (MTD) of Romidepsin in addition to CHOEP-21 and to test the safety and feasibility of CHOEP-21 in combination with dose escalation of Romidepsin (8, 10, 12, 14 mg). The dose level defined as MTD of Romidepsin will be used for the subsequent phase II study.<br>2. A phase II study to evaluate the efficacy (response rate, progression free survival and overall survival) and safety of Ro-CHOEP-21 incorporated into a treatment strategy including SCT.
**INDICATION**	Newly diagnosed patients with Peripheral T-cell lymphomas including: Peripheral T-cell lymphomas not otherwise specified (PTCL-NOS), Angioimmunoblastic T-cell lymphoma (AITL) and ALK negative Anaplastic large-cell lymphoma (ALCL).
**OBJECTIVES PHASE I**	**Primary:**<br>• To define the maximum tolerated dose (MTD) of Ro-CHOEP-21<br><br>**Secondary:**<br>• To assess the feasibility of the Ro-CHOEP-21 treatment strategy combined with SCT
**OBJECTIVES PHASE II**	**Primary:**<br>• To evaluate the efficacy in term of Progression Free Survival (PFS) of Ro-CHOEP-21<br><br>**Secondary:**<br>• To evaluate ORR and in particular CR rate achieved before and after SCT.<br>• To evaluate event free survival (EFS) and overall survival (OS)<br>• To evaluate the safety of treatment<br>• To evaluate the outcome of early allogeneic SCT in patients in PR at the end of induction phase<br>• To estimate the treatment-related mortality (TRM)<br>• To evaluate the incidence of acute and chronic GVHD in allografted patients<br>• To improve the knowledge on PTCL diagnosis, classification and biology.<br><br>**Exploratory:**<br>• Evaluation of response biomarkers (eg TET2 mutations)
**NUMBER OF PLANNED PATIENTS**	**Phase I**: 21-24 patients (estimated 50% treated at the MTD)<br>**Phase II**: 110 patients in total, including the patients (approximately 9-15) expected from the phase I study (treated at the MTD)
**NUMBER OF CENTER**	**40**

**ELECTION CRITERIA**	**INCLUSION CRITERIA**<br>1. age ≥18 e ≤ 65 years<br>2. Peripheral T-cell lymphomas at diagnosis including: PTCL-NOS, AITL, ALK negative ALCL<br>3. Stage II-IV<br>4. Written informed consent<br>5. No prior treatment for lymphoma<br>6. No Central Nervous System (CNS) disease (meningeal and/or brain involvement by lymphoma)<br>7. HIV negativity<br>8. Absence of active hepatitis C virus (HCV) infection<br>9. HBV negativity or patients with HBcAb +, HBsAg -, HBs Ab+/- with HBV-DNA negativity (in these patients Lamivudine prophylaxis is mandatory)<br>10. Levels of serum bilirubin, alkaline phosphatase and transaminases < 2 the upper normal limit, if not disease related<br>11. No psychiatric illness that precludes understanding concepts of the trial or signing informed consent<br>12. Ejection fraction > 50% and myocardial stroke in the last year nor QT prolongation (QTc interval < 480 msec using the Fridericia formula)<br>13. Clearance of creatinine > 60 ml/min if not disease related<br>14. Spirometry Diffusion Capacity (DLCO) > 50%<br>15. Absence of active, uncontrolled infection<br>16. For males and females of child-bearing potential, agreement upon the use of effective contraceptive methods prior to study entry, for the duration of study participation and in the following 90 days after discontinuation of study treatment<br>17. Availability of histological material for central review and pathobiological studies.<br><br>**EXCLUSION CRITERIA**<br>1. age <18 e > 65 years<br>2. Hystology other than: PTCL-NOS, AITL, ALK negative ALCL<br>3. Stage I<br>4. Prior treatment for lymphoma<br>5. Positive serologic markers for human immunodeficiency virus (HIV)<br>6. Active hepatitis B virus (HBV) infection<br>7. Active hepatitis C virus (HCV) infection<br>8. Levels of serum bilirubin, alkaline phosphatase and transaminases > 2 the upper normal limit, if not disease related<br>9. Ejection fraction < 50% and no myocardial stroke in the last year or QT prolongation (QTc interval > 480 msec using the Fridericia formula)<br>10. Clearance of creatinine < 60 ml/min if not disease related<br>11. Spirometry Diffusion Capacity (DLCO) < 50%<br>12. Pregnancy or lactation<br>13. Patient not agreeing to take adequate contraceptive measures during the study<br>14. Psychiatric disease that precludes understanding concepts of the trial or signing informed consent<br>15. Any active, uncontrolled infection

16. Prior history of malignancies other than PTCLs in the last five years (except for basal cell or squamous cell carcinoma of the skin or carcinoma in situ of the cervix or breast).

TREATMENT PLAN PHASE I

A1) **Induction phase**		
Ro-CHOEP-21 x 3 cycles		
	Romidepsin (dose escalation)	*Starting dose: 12mg/ms* iv day +1 and +8<br>*Dose modification according to toxicity:*<br>• *14mg/ms day +1 and +8*<br>• *10mg/ms day +1 and +8*<br>• *8mg/ms day +1 and +8*
	CHOEP-21	• *Doxorubicin 50 mg/ms* iv *day +1,*<br>• *Vincristin 1.4 mg/ms (maximum 2.0 mg total dose)* iv *day+1,*<br>• *Cyclophosphamide 750 mg/ms* iv *day +1,*<br>• *Etoposide 100mg/ms* iv *from day +1 to +3*<br>• *Prednisone100 mg orally from days +1 to +5*
According to the response achieved after the first 3* Ro-CHOEP-21 *cycles:		
PR or CR	Ro-CHOEP-21 for 3 additional cycles followed by phase_A2	
SD or PD	Treatment failures, proceed to salvage according to each institutional policy	

A2) **Stem cell mobilization and transplantation phase**		
	Response evaluation and one DHAP course followed by peripheral stem cell harvesting	• *Dexamethasone 40mg* iv *day +1 +2 +3 +4*<br>• *Cisplatin 100mg/ms* iv *day +1*<br>• *Cytarabine 2gr/ms bid* iv *day +2(in-patient version) or Ara-C 2 gr/ms* iv *day +2 and day +3 (out-patient version)*<br>• *G-CSF 5 μcg/kg/day* sc *starting from day +5 until peripheral blood stem cell harvest*
According to response achieved after 6 Ro-CHOEP-21 cycles:		
CR	BEAM or FEAM or CEAM followed by auto-SCT	• *BCNU 300 mg/ms* iv *day -7 (or Fotemustine150 mg/ms* iv *day -7, -6 or 300mg/mq day -7; or CCNU 200 mg/ms iv day -7)*<br>• *Etoposide 200 mg/ms* iv *day -6,-5,-4, 3*<br>• *Cytarabine 200 mg/mq bid* iv *day -6,-5,-4, -3*<br>• *Melphalan140 mg/ms* iv *day-2*
PR	• **Allogeneic SCT** with HLA-identical (A, B, C, DR, DQ loci) or one antigen mismatched (class I) sibling donors. Donor selection is	• *Thiotepa 15mg/kg (5mg/kg every 12 hours for 3 doses* iv *on day –6 and -5)*<br>• *or Thiotepa 10mg/kg(5mg/kg*

<table>
<tr><td></td><td></td><td>based on molecular high-resolution typing (4 digits) of the HLA gene loci class I (HLA-A, B, and C) and class II (DRB1, DQB1). In case, no class I and class II completely identical urelated donor (10 out of 10 gene loci) can be identified, the degree of histocompatibility between patient and donor must fulfill with the minimal degree of matching established by the Italian Bone Marrow Donor Registry: HLA-A and HLA-B antigen histocompatibility and HLA-DRB1 allelic histocompatibility.

• when a suitable donor is not available: BEAM or FEAM or CEAM followed by Auto-SCT</td><td>every 12 hours for 2 doses iv on day -5) if age >55yrs or Hematopoietic Cell Transplant-Comorbidity Index≥2)
• Cyclophosphamide 30mg/kg iv day -4, -3
• Fludarabine 30mg/mg iv day-4, -3
• GvHD prophylaxis: cyclosporine and short course methotrexate</td></tr>
<tr><td></td><td>< PR</td><td colspan="2">Treatment failures, proceed to salvage according to each institutional policy</td></tr>
<tr><td rowspan="9">TREATMENT PLAN PHASE II</td><td colspan="3"><u>A1) Induction phase</u></td></tr>
<tr><td colspan="3">Ro-CHOEP-21 x 3 cycles</td></tr>
<tr><td></td><td>Ro-CHOEP-21</td><td>• Romidepsin dose according to phase I iv day +1 and +8
• Doxorubicin 50 mg/ms iv day +1,
• Vincristin 1.4 mg/ms (maximum 2.0 mg total dose) iv day+1,
• Cyclophosphamide 750 mg/ms iv day +1,
• Etoposide 100mg/ms iv from day +1 to +3
• Prednisone100 mg orally from days +1 to +5</td></tr>
<tr><td colspan="3">According to the response achieved after the first 3 Ro-CHOEP-21 cycles:</td></tr>
<tr><td>PR or CR</td><td>Ro-CHOEP-21 for 3 additional cycles followed by phase_A2</td><td></td></tr>
<tr><td>SD or PD</td><td colspan="2">Treatment failures, proceed to salvage according to each institutional policy</td></tr>
<tr><td colspan="3"><u>A2) Stem cell mobilization and transplantation phase</u></td></tr>
<tr><td></td><td>Response evaluation one DHAP course followed by peripheral stem cell harvesting</td><td>• Dexamethasone 40mg iv day +1 +2 +3 +4
• Cisplatin 100mg/ms iv day +1
• Cytarabine 2gr/ms bid iv day +2(in-patient version) or Ara-C 2 gr/ms iv day +2 and day +3 (out-patient version)
• G-CSF 5 μcg/kg/day sc starting from day +5 until peripheral blood stem cell harvest</td></tr>
<tr><td colspan="3">According to response achieved after 6 Ro-CHOEP-21cycles:</td></tr>
<tr><td></td><td>CR</td><td>BEAM or FEAM or CEAM followed by Auto-SCT</td><td>• BCNU 300 mg/ms iv day -7 (or Fotemustine150 mg/ms iv</td></tr>
</table>

		day -7, -6 or 300mg/mq day -7; or CCNU 200 mg/ms iv day -7)<br>• *Etoposide 200 mg/ms* iv *day -6,-5,-4, 3*<br>• *Cytarabine 200 mg/mq bid* iv *day -6,-5,-4, -3*<br>• *Melphalan140 mg/ms* iv *day-2*
PR	• **Allogeneic SCT** with HLA-identical (A, B, C, DR, DQ loci) or one antigen mismatched (class I) sibling donors. Donor selection is based on molecular high-resolution typing (4 digits) of the HLA gene loci class I (HLA-A, B, and C) and class II (DRB1, DQB1). In case, no class I and class II completely identical unrelated donor (10 out of 10 gene loci) can be identified, the degree of histocompatibility between patient and donor must fulfill with the minimal degree of matching established by the Italian Bone Marrow Donor Registry: HLA-A and HLA-B antigen histocompatibility and HLA-DRB1 allelic histocompatibility.<br><br>• when a suitable donor is not available: BEAM or FEAM or CEAM followed by Auto-SCT	• *Thiotepa 15mg/kg* iv *(5mg/kg every 12 hours for 3 doses on day –6 and -5)*<br>• *or Thiotepa 10mg/kg* iv *(5mg/kg every 12 hours for 2 doses on day -5) if >55 yrs or Hematopoietic Cell Transplant-Comorbidity Index≥2) day -5*<br>• *Cyclophosphamide 30mg/kg* iv *day -4, -3*<br>• *Fludarabine 30mg/mg* iv *day-4, -3*<br>• *GvHD prophylaxis: cyclosporine and short course methotrexate*
< PR	Treatment failures, proceed to salvage according to each institutional policy	

STUDY PROCEDURES

Staging evaluation, baseline

Baseline assessment must be performed during 30 days before starting therapy.

- Complete medical history, ECOG performance status, physical examination, vital signs
- ECG with QTc calculation and echocardiogram or MUGA scan for LVEF evaluation
- Spirometry DLCO
- Complete blood count, hematology workup and biochemistry
- HBsAg, HBcAb, HCV and HIV serology
- Lymph-node or tissue biopsy for histological diagnosis and shipment of paraffin block for centrally pathology review and for biological studies
- Aspirate and bone marrow biopsy
- CT scan neck, chest, abdomen and pelvis
- Total body PET scan
- Pregnancy test (if applicable)
- Lumbar puncture
- Written informed consent
- If clinically indicated: neurological visit, RMN brain/spine, GI endoscopy, ORL visit
- HLA typing (not mandatory)

Evaluation at each Ro-CHOEP courses

	- Blood count and complete workup with biochemistry, physical examination, vital signs and hematological and extra-hematological toxicity evaluation the day before or day 1 and day 8 of therapy and between two cycles and during aplasia phase and/or till granulocytes and platelets recovery.<br>- Electrocardiogram will be performed just before romidepsin infusion (after administration of antiemetic premedication if possible) at day 1 of each cycle for measurement of corrected QT interval according to the Fridericia formula and in case of cardiac event or clinical signs compatible with heart rhythm disorder and in case of biological abnormalities.<br><br>***Intermediate response evaluation***<br>The evaluation of intermediate response will be assessed after 3 courses of Ro-CHOEP-21.<br>- ECOG performance status, physical examination, vital signs<br>- Blood count and complete workup with biochemistry<br>- Aspirate and bone marrow biopsy (if positive at baseline)<br>- CT scan neck, chest, abdomen and pelvis<br>- Total body PET scan (not mandatory)<br>- HLA typing and start donor search mandatory for patients in PR<br><br>Responsive patients (in partial or complete response) after three cycles of therapy, will continue the trial and will be treated with 3 more courses of Ro-CHOEP as planned.<br><br>***Post-Induction evaluation***<br>The evaluation post induction will be assessed after six courses of Ro-CHOEP.<br>- ECOG performance status, physical examination, vital signs<br>- Blood count and complete workup with biochemistry<br>- Peripheral blood samples for biological studies<br>- Aspirate and bone marrow biopsy (if positive at baseline)<br>- CT scan neck, chest, abdomen and pelvis<br>- Total body PET scan<br><br>Patients in CR will receive one course of DHAP followed by peripheral stem cells harvesting and BEAM or FEAM or CEAM followed by autologous stem cell transplant; patients in PR will receive one course of DHAP followed by peripheral stem cells harvesting and allo-SCT; patients in PR when a suitable donor is not available, will receive one course of DHAP + PBSC harvest followed by BEAM or FEAM or CEAM followed by autologous stem cell transplant; patients in SD or progressive disease will receive salvage treatment according to each institutional policy outside the protocol. In patients in PR when a suitable donor is available, DHAP plus PBSC harvest can be avoided.<br><br>***Post –SCT evaluation***<br>Final evaluation will be performed one-two months after the end of SCT.<br>- ECOG performance status, physical examination, vital signs<br>- Blood count and complete workup with biochemistry<br>- Aspirate and bone marrow biopsy (if positive at baseline)<br>- CT scan neck, chest, abdomen and pelvis

	- Total body PET scan<br>Complete response, Partial response or no response will defined according to Lugano 2014 response criteria.<br><br>***Follow-up***<br>The total duration of follow-up is 5 years with the following plan: every 3 months during the first year after chemotherapy and then every 6 months up to 3 years after chemotherapy and then annually for a further 2 years. At all these steps will be evaluated:<br>- ECOG performance status, physical examination, vital signs<br>- Blood count and complete workup with biochemistry<br>- CT scan neck, chest, abdomen and pelvis<br>- Total body PET scan when clinically indicated
**STATISTICAL CONSIDERATIONS:**	***PHASE I STUDY***<br><br>**Endpoints**<br>**Primary endpoint**<br>• Incidence of dose-limiting toxicity (DLT) of Ro-CHOEP-21, considering as maximum dose the one causing induction of any grade ≥ 3 non hematologic toxicity or a delay >15 days of planned cycle date observed during the first two cycles according to the definitions of NCI Common Terminology Criteria for Adverse Events (CTCAE), version 4.0 (2009)<br><br>**Secondary endpoints**<br>• Proportion of patients reaching SCT<br>• Overall response rate (ORR, defined according to the Lugano Classification 2014 response criteria) of the combination of Ro-CHOEP-21.<br><br>**Statistical design**<br>The continual reassessment method (CRM) for dose-finding phase I study (Zohar, 2001; O'Quigley and Zohar, 2006) will be used as the dose allocation rule in the trial for groups of three patients at each dose. The design of this dose-finding phase clinical trial is chosen to assess the maximum tolerated dose (MTD) of romidepsin when administered in combination with CHOEP chemotherapy in the treatment of patients with T-cell lymphoma, candidate to stem cell transplant. The MTD is defined as the dose that achieves a dose-limiting toxicity (DLT) in 33% of patients. Four dose levels are tested, namely 8, 10, 12 and 14 mg/sqm. The CRM method is based on a mathematical modelling of dose–DLT relationship, iteratively updated using Bayes theorem along the trial, as follows. First, before trial onset, prior opinions about DLT probability at each dose level are elicited from expert clinicians on the basis of their personal experience and on literature. These initial guesses, which relied on the opinion of participating clinicians, were fixed at 0.15, 0.20, 0.25, and 0.30, respectively. The uncertainty in this dose–DLT relationship is incorporated into a prior. Then, the first three included patients are administered the third dose level (12 mg/ms). After the enrollment of the first three patient, accrual continues, with grouped inclusions of three patients per dose level. Then, on the basis of observed responses (DLT or not), DLT probabilities of all dose levels are updated using Bayes theorem. The dose level associated with an updated DLT probability close to 33% is recommended

	to be administered to the next patient cohort. All this process is re-run until the fixed sample size (N=24) is reached, or in case of fulfilled stopping criteria measuring futility of trial continuation (Zohar, 2003).<br><br>**<u>Study design and treatment</u>**<br>The study consists of the following consecutive phases: A1) Induction phase and A2) stem cell mobilization and transplantation phase. Newly diagnosed patients will receive induction treatment with Romidepsin in combination with CHOEP-21 for 3 cycles, CR or PR patients will receive 3 additional courses, while not responders will be switched to an early salvage treatment and censored as a failure. To define the maximum tolerated dose (MTD), four dose levels of Romidepsin will be tested. The dose of Romidepsin will be modulated according to continual reassessment method. The starting dose for the first three patients will be 12 mg/ms (based on expert opinion). Stem cell mobilization will be with a DHAP course followed by G-CSF. At the end of induction, patients in CR/CRu will receive auto-SCT and patients in PR allo-SCT (if a donor is available).<br><br>***<u>PHASE II STUDY</u>***<br><br>**<u>Endpoints</u>**<br>**Primary endpoint**<br>• PFS on intention to treatment (ITT) evaluated at 18 months. PFS will be defined as the time between the date of enrolment and the date of disease progression, relapse or death from any cause.<br><br>**Secondary endpoints**<br>• ORR and CR (defined according to the Lugano Classification 2014 response criteria), after Ro-CHOEP-21 and after SCT Event free survival (EFS) induction treatment and after SCT<br>• Event free survival (EFS) defined as the time between the date of enrollment and the date of discontinuation of treatment for any reason<br>• Overall survival (OS) defined as the time between the date of enrolment and the date of death from any cause in the ITT population enrolled in the study<br>• PFS and OS in patients not responding to the first 3 courses of Ro-CHOEP-21<br>• Evaluation during the interim analyses of any grade III or higher toxicities, recorded and classified according to the definitions of NCI Common Terminology Criteria for Adverse Events (CTCAE), version 4.0 (2009)<br>• Evaluation during all the pretransplant phase of any grade III or higher toxicities, recorded and classified according to the definitions of NCI Common Terminology Criteria for Adverse Events (CTCAE), version 4.0 (2009)<br>• Any grade III or higher toxicities, recorded and classified according to the definitions of NCI Common Terminology Criteria for Adverse Events (CTCAE), version 4.0 (2009)<br>• Treatment-related mortality defined as any death that was not attributable to the lymphoma.<br>• Incidence of acute and chronic GVHD in allografted patients

	**Exploratory endpoint**<br>• Evaluation of response biomarkers (eg TET2 mutations)<br><br>**<u>Statistical design</u>**<br>The sample size of this single arm, phase II trial, has been calculated using the PFS as the primary endpoint, according to the two-stage design proposed by Case and Morgan (2003), without interim pause in the enrolment. According to available evidence, the 1.5 year PFS of newly diagnosed PTCL patients treated with anthracycline based therapy (CHOP/CHOEP), followed by auto-SCT, is around 55% (null hypothesis, H0). With our experimental strategy, based on 6 courses of CHOEP-21 plus Romidepsin, followed by SCT in chemosensitive disease, we hypothesize to achieve an overall 1.5 year PFS of 70% (alternative hypothesis, H1).<br>To demonstrate an absolute improvement from 55% (literature data) to 70% of the 1.5 year PFS, with an alpha error of 0.05 (one tail), a beta error of 0.10, and assuming 3 years of constant accrual and at least 1.5 years of follow-up after the enrolment of the last patient, the required total sample size calculated to minimize the ETSL (expected total study length) is 110 (sample size calculated with the Sample Size Tables for Clinical Studies, 3rd edition, by Machin et al, 2009).<br>With this design the interim analysis will be performed when the first 75 patients have been enrolled. At this time the Kaplan-Meier 1.5 years PFS will be estimated, with its standard error, to calculate the Z interim test. To proceed with the enrolment, the threshold of the Z interim test for efficacy should be at least 0.650.<br>If this case, 35 further patients will be enrolled to reach the planned total sample size of 110 and the final analysis will be performed after the last enrolled patient has been followed for 18 months. To reject the null hypothesis the threshold of the Z final statistic must be greater than 1.522.
**TIMING**	Duration of accrual: 3 years.<br>Duration of treatment 6-8 months<br>Duration of follow up: 5 years.

2 FLOW CHART

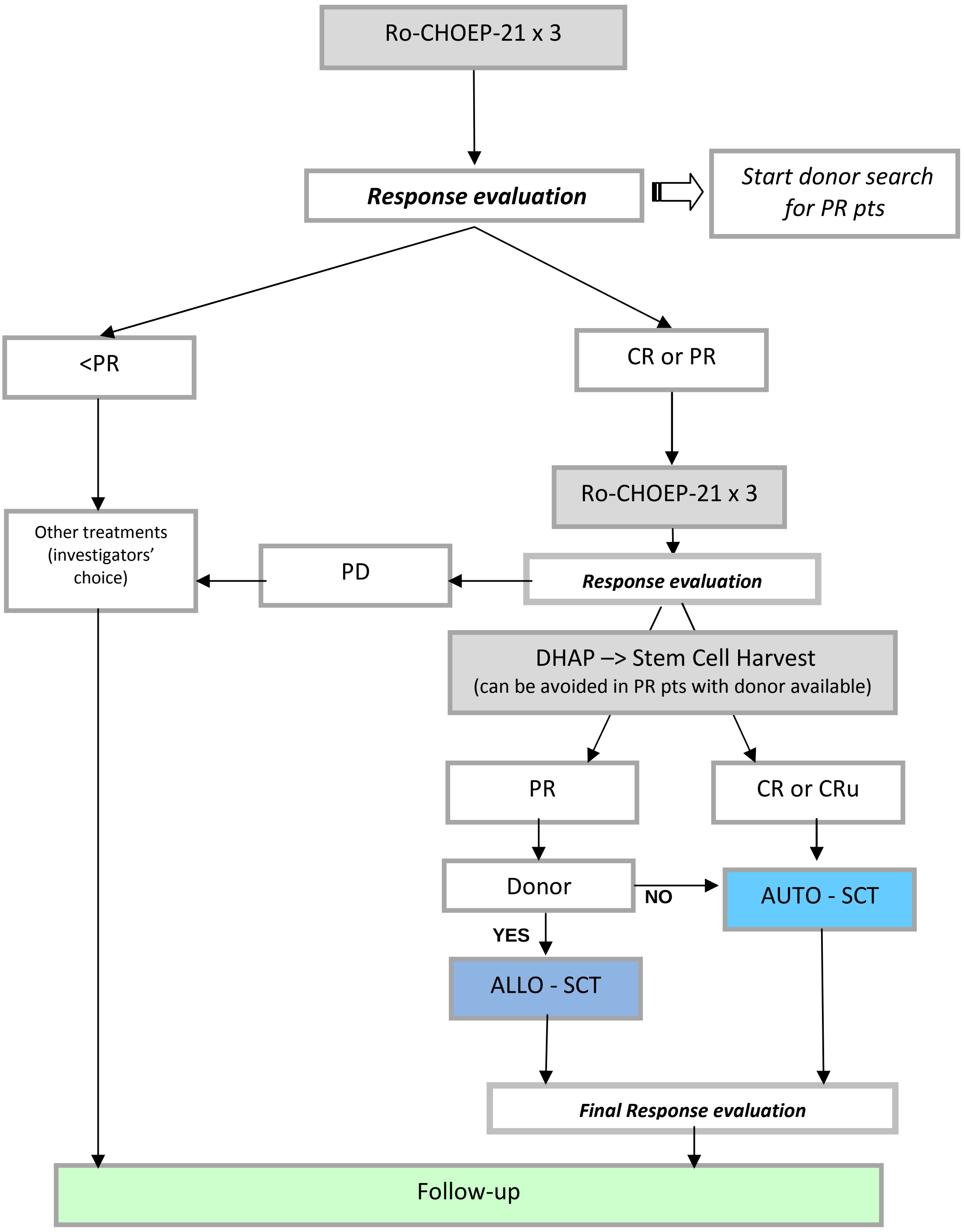

Ro-CHOEP-21 x 3
Response evaluation
Start donor search for PR pts
<PR
CR or PR
Ro-CHOEP-21 x 3
Other treatments (investigators' choice)
PD
Response evaluation
DHAP –> Stem Cell Harvest
(can be avoided in PR pts with donor available)
PR
CR or CRu
Donor
NO
AUTO - SCT
YES
ALLO - SCT
Final Response evaluation
Follow-up

3 BACKGROUND AND INTRODUCTION

3.1 Study background

Peripheral T-cell lymphomas (PTCLs) represent approximately 10-15% of lymphoid neoplasms in Western countries. PTCLs are a heterogeneous group of tumours, extremely difficult to classify by morphology only. According to the WHO classification of Tumors of the Hematopoietic and Lymphoid Tissues, each entity should be defined by combining morphologic, immunophenotipic, genetic and clinical features[1]. This diagnostic approach enables to distinguish two major subgroups: PTCL specified and not otherwise specified (NOS). The most common (~25%) and heterogeneous subtypes are PTCL-NOS, followed by Angioimmunoblastic T-cell Lymphoma (AITL), Anaplastic Lymphoma Kinase-positive (ALK^+) and Anaplastic Lymphoma Kinase-negative (ALK^-) Large Cell Lymphoma (ALCL). The REAL classification, largely adopted by the WHO classification for lymphomas, updated in 2008, provides useful definitions for the diagnosis of the major subtypes of PTCL. However, the diagnosis of PTCL is still challenging and requires expert pathologists due to the relative low frequency of the disease and the lack of unique distinctive features.

PTCLs, with the exception of ALK^+ALCL, most commonly occurring in middle-aged to elderly patients, present in advanced stage and are associated with unfavourable clinical characteristics. Compared to B-cell lymphomas (B-NHL), PTCLs, with the exception of ALK^+ALCL and primary cutaneous ALCL, carry universally poor outcome; whereas the prognosis of B-cell lymphomas has changed due to the increasing number of new drugs and therapeutic approaches available, T-cell lymphomas remain an adverse niche in the lymphoproliferative scenario.

Historically, PTCLs were treated similarly to aggressive B-NHL with anthracycline-based combination chemotherapy like CHOP (cyclophosphamide, doxorubicin, vincristine, prednisone) with disappointing results. When the main subgroups of T-cell lymphomas were analyzed, only ALK^+ALCL had an equivalent or superior prognosis compared to diffuse large B cell Lymphoma (DLBCL). Neither intensified/escalated chemotherapeutic approaches [2-3], nor the addition of monoclonal antibodies such as Alemtuzumab [4], have demonstrated a clear advantage in remission rate and survival. The discouraging results achieved with conventional therapies led to investigate new concepts, including high dose chemotherapy (HD) followed by autologous stem cell transplantation (auto-SCT) or allogeneic stem cell transplantation (allo-SCT).

So far no randomized trials have evaluated the role of auto-SCT, neither frontline nor as salvage therapy. However, some phase II prospective studies have specifically investigated the role of frontline auto-SCT in PTCLs. Across the studies, there are few recurrent points that emerge:
a) The procedure is safe and feasible, with a transplant related mortality (TRM) of less than 5%;
b) approximately 25% of patients do not reach the transplant phase mainly because of refractory or progressive disease;
c) Only chemosensitive disease can benefit from auto-SCT;
d) The achievement of complete response (CR) before SCT is recognized as a prerequisite for long-term disease control;
e) Long term overall survival (OS) for patients with chemosensitive disease before auto-SCT is approximately 50%.

Of note, based on the encouraging results reported by the German study on the addition of etoposide (E) to the CHOP regimen (CHOEP)[5], the Nordic group[6] designed a phase II study to evaluate the impact of a dose-intensified induction schedule (CHOEP-14 for 6 cycles) consolidated in first PR/CR with high-dose therapy (BEAM/auto-SCT). After induction, 82% of patients were in CR or PR. Early refractory disease to induction treatment was observed in 16% of patients. With an average follow up of 60.5 months, 5-year OS and PFS were 51% and 44%, respectively. The encouraging outcome achieved with dose/dense CHOEP followed by auto-SCT, suggests that this strategy is probably the best treatment currently available.

The role of allo-SCT in aggressive NHL is still under investigation. Allo-SCT provides several advantages over auto-SCT, including a lymphoma-free graft and a potentially active graft-versus-lymphoma (GVL) effect. However very few studies have specifically addressed the role of allo-SCT in the setting of PTCLs. Allo-SCT has been mainly used in PTCLs patients relapsed after auto-SCT or with refractory disease. Overall, retrospective studies on myeloablative regimens, while highlighting concern on toxicity and TRM, confirmed a potential role for allo-SCT as salvage treatment in aggressive lymphomas and demonstrated a lower relapse rate compared to auto-SCT [7-9]. However comparative studies of auto-SCT versus allo-SCT failed to demonstrate a survival benefit in the allografting group due to the higher TRM [9]. Recently, reduced-intensity conditioning (RIC) regimens have been offered as an alternative to mieloablative ones in order to reduce organ toxicity and thus TRM [10-11]. Across the retrospective and prospective studies on relapsed/refractory PTCLs two important points emerge:
a) TRM with RIC regimens is approximately 20 - 25%;
b) long term OS for chemosensitive relapses is around 40%.
Based on the encouraging results shown by RIC-SCT in the salvage setting, a national phase II trial[12] was designed in Italy in order to evaluate the role of frontline treatment intensification in PTCLs. An intensified program including CHOP-alemtuzumab followed by methotrexate, cytarabine, cyclophosphamide and consolidation with either auto-SCT or allo-STC, based on genetic stratification, was conducted. Sixty percent of patients had a chemosensitive disease before transplant (51% in CR and 9% in PR) while 32% were non-responders. With a median follow up of 31 months, 49% patients were in CR, 38% died due to progressive disease, 13% died of toxicity. The estimated 4-year OS and PFS were 43% and 47%, respectively. At the moment no differences are observed between auto-SCT and allo-SCT in term of PFS and OS. From this study few suggestions emerge: a) primary refractory or early progressive patients do not respond to a more aggressive chemotherapeutic approach, therefore alternative strategies are needed to rescue this subgroups of patients, b) chemosensitive patients who received consolidation with SCT had a superior outcome when compared to historical data based on chemotherapy only.

With regard to novel treatment options the are two molecules pralatrexate and romidepsin. For type of admistration and toxicity profile pralatrexate cannot be combined with other multiagent chemotherapy. On the other hand, promising results have been reported in cutaneous T cell lymphoma (CTCL) with Romidepsin, a histone deacetylase inhibitor (HDAC), that inhibits class I and class II enzymes. Preclinical studies on T cell lymphoma have reported a potent anti-tumor activity. In 2009, a phase I study on advanced stage CTCL treated with Romidepsin (D1-D8-D15 every 28 days) until progressive disease showed a ORR of 34% with an average duration of response of 13.7 months[13]. The most common adverse effects were fatigue, nausea, vomiting, anorexia, and transient thrombocytopenia and neutropenia. Romidepsin was therefore tested also in

pretreated PTCLs. In a phase 2 trial, Romidepsin in monotherapy showed an ORR of 25%, including 15% with CR/CRu, with a median response duration of 17 months, confirming clinically activity also in PTCL [14]. Based on these observations, Romidepsin has been tested in combination with CHOP[15] in untreated PTCLs. The dose of 12mg/ms was identified as feasible with manageable hematological toxicity.

3.2 Rational of the study

New therapeutic options are needed to overcome the poor prognosis of PTCL patients. As previously described, consolidation with SCT can be considered a standard strategy in young patients with chemosensitive disease. However, 25-30% of patients do not become transplant eligible due to primary refractory or early progressive disease.

The reduction of refractory or early progressive disease is an unmet clinical need. Currently, CHOEP represents the best treatment option in preparation to auto-SCT. To increase the response rate, we will use CHOEP in combination with Romidepsin (Ro-CHOEP), a non cross resistant agent that showed anti tumor activity in T-cell lymphomas and a manageable toxicity profile in combination with CHOP chemotherapy.

To date, the role of allo-SCT vs auto-SCT in the upfront setting has not been clarified. Moreover, no conclusive data are available regarding the impact of the quality of response before SCT and we currently do not known if achieving CR versus PR before SCT can significantly improve long term outcome. In general, all studies showed that patients in CR at transplant had a better long term outcome. In our previous national pilot study, CR before transplant, was an independent prognostic factor for long term survival, suggesting that auto-SCT could be the treatment of choice for this setting of patients. In the present study, all responder patients (≥ PR) will proceed with SCT as consolidation treatment. Patients will be stratified to received auto-SCT versus allo-SCT according to the response achieved after induction treatment (CR vs PR). Patients achieving CR will proceed with auto-SCT. Allo-SCT will be offered to patients assuming the need for further intensification. There is evidence suggesting that chemo-refractory disease does not respond to high-dose thus, for early treatment failure, alternative strategies should be investigated in preparation to SCT. For this reason, patients achieving less than PR, after 3 cycles of induction treatment or at the end of the induction, will not proceed with SCT and will be considered treatment failures.

To date, the limited number of patients enrolled in clinical trials and the marked heterogeneity of the histological PTCLs subgroups do not allow to provide reliable subtype-specific information to drive therapeutic decisions. The knowledge of PTCL biology is modest, the classification difficult and there are no reliable response biomarkers . For these reasons, this trial includes a central revision by an expert pathologist and a centralized sample collection in order to perform biological studies. Both the confirmation of diagnosis and the collection of biological samples will allow studies to increase the knowledge on PTCL classification and biology.

3.3 Romidepsin

Romidepsin (ISTODAX®; Celgene, Summit, NJ) is a natural product obtained from the bacteria Chromobacterium violaceum. It is a structurally unique, potent, bicyclic class 1 selective histone deacetylase (HDAC) inhibitor. HDAC inhibitors have been shown to induce the acetylation of both histones and other proteins [16, 17], resulting in antitumor activity due to increased tumor suppressor gene transcription, growth inhibition, cell cycle regulation, and apoptosis [18-22]. This compound was approved in 2009 by FDA for the treatment of cutaneous T-cell lymphoma (CTCL) in patients who had received at least one prior systemic therapy. Two non-comparative, multicentres, phase II trials were conducted in patients with relapsed, refractory or advanced CTCL studying the effects of Romidepsin administered intravenously at 14 mg/ms as 4 hr infusion on days 1, 8, 15 every 4 weeks were performed [13, 23]. In both trials, therapy with Romidepsin was associated with an overall response rate (ORR), including complete and partial response (CR and PR), of 34% and a CR of 6%. In 2011, FDA approved Romidepsin for the treatment of patients with relapsed or refractory PTCL who have received at least one prior systemic therapy. In this setting of patients, Romidepsin administered with the same dose/schedule used in CTCL phase II trials, induced 25% (i.e., 33 out of 130 treated patients) objective response rate (ORR represented by complete and partial responses), including 19 patients (15%) with complete response (CR) and complete response with incomplete blood count recovery (CRu) defined by an Independent Review Committee, and durable responses (14). Adverse events (AEs) associated to Romidepsin treatment were manageable, consistent with other HDAC inhibitors and include: GI disturbances (vomiting, nausea, diarrhea abdominal pain, constipation and stomatis), hematologic abnormalities (thrombocytopenia, leucopenia and anemia), asthenia/fatigue and infections. The most frequent non hematological drug-related AEs were nausea and vomiting, which were primarily Grade 1-2 and did not result in drug discontinuation. Grade 3-4 asthenia or fatigue was reported in approximately 10% of patients. Hematologic abnormalities represented the most common adverse events of Grade 3-4 severity observed in patients with both CTCL and PTCL due to disease involvement in bone marrow and prior myelosuppressive chemotherapeutic regimens. Several treatment-emergent morphological changes in ECGs (including T-wave and ST-segment changes) have been reported in clinical studies. The clinical significance of these changes is unknown. Cautionary patients with congenital long QT syndrome, a history of significant cardiovascular disease, and patients taking medicinal products that lead to significant QT prolongation should be straightly monitored for cardiac function.

3.4 Romidepsin Safety Profile

3.4.1 *Identified and Potential Risks of Romidepsin*

- Monotherapy

The following list summarizes treatment-emergent adverse events that occurred at an incidence of greater than 2% among patients receiving romidepsin monotherapy, by indication and MedDRA SOC and preferred term (n=891). Overall, the rate of adverse events was higher in patients with hematologic malignancies, including T-cell lymphomas (437 of 447 patients; 98%) than those with solid tumors (330 of 444 patients; 74%). Review of adverse events by system organ class (SOC) showed that particular types of adverse events generally occurred at a higher incidence in patients with hematologic malignancies than those with solid tumors, including gastrointestinal disorders

(79% versus 62%, respectively), general disorders and administration site conditions (76% versus 55%, respectively), blood and lymphatic system disorders (64% versus 45%, respectively), infections and infestations (50% versus 12%, respectively), and skin and subcutaneous tissue disorders (33% versus 12%, respectively). Although the incidence of adverse events was higher in patients with hematologic malignancies than those with solid tumors, the particular types of adverse events reported were generally similar by indication.

Table 1. Treatment-emergent Adverse Events Reported by > 2% of Patients Receiving Romidepsin Monotherapy, by Indication and MedDRA SOC and Preferred Term, for All Adverse Events and Grade 3 and Grade 4 Adverse Events (N = 891)

	Indication					
	Hematologic Malignancies (N = 447)			Solid Tumors (N = 444)		
MedDRA SOC/Preferred Term	All AEs n (%)	Grade 3 AEs n (%)	Grade 4 AEs n (%)	All AEs n (%)	Grade 3 AEs n (%)	Grade 4 AEs n (%)
At least 1 TEAE[a]	437 (97.8)	281 (62.9)	102 (22.8)	330 (74.3)	200 (45.0)	33 (7.4)
**Blood and Lymphatic System Disorders**	**287 (64.2)**	**161 (36.0)**	**49 (11.0)**	**198 (44.6)**	**50 (11.3)**	**2 (0.5)**
Anaemia	177 (39.6)	52 (11.6)	3 (0.7)	141 (31.8)	24 (5.4)	2 (0.5)
Thrombocytopenia	157 (35.1)	49 (11.0)	13 (2.9)	29 (6.5)	2 (0.5)	0
Neutropenia	130 (29.1)	48 (10.7)	29 (6.5)	12 (2.7)	2 (0.5)	0

MedDRA SOC/Preferred Term	Indication					
	Hematologic Malignancies (N = 447)			Solid Tumors (N = 444)		
	All AEs n (%)	Grade 3 AEs n (%)	Grade 4 AEs n (%)	All AEs n (%)	Grade 3 AEs n (%)	Grade 4 AEs n (%)
Febrile Neutropenia	22 (4.9)	19 (4.3)	1 (0.2)	2 (0.5)	2 (0.5)	0
**Cardiac Disorders**	**75 (16.8)**	**9 (2.0)**	**3 (0.7)**	**37 (8.3)**	**8 (1.8)**	**2 (0.5)**
Tachycardia	15 (3.4)	0	0	6 (1.4)	0	0
Sinus Tachycardia	13 (2.9)	0	0	5 (1.1)	2 (0.5)	0
**Eye Disorders**	**33 (7.4)**	**4 (0.9)**	**0**	**30 (6.8)**	**2 (0.5)**	**0**
Vision Blurred	8 (1.8)	0	0	10 (2.3)	1 (0.2)	0
**Gastrointestinal Disorders**	**354 (79.2)**	**50 (11.2)**	**6 (1.3)**	**277 (62.4)**	**52 (11.7)**	**4 (0.9)**
Nausea	293 (65.5)	19 (4.3)	0	227 (51.1)	28 (6.3)	0
Vomiting	184 (41.2)	19 (4.3)	1 (0.2)	162 (36.5)	31 (7.0)	0
Diarrhoea	124 (27.7)	7 (1.6)	1 (0.2)	63 (14.2)	5 (1.1)	0
Constipation	120 (26.8)	5 (1.1)	0	101 (22.7)	5 (1.1)	1 (0.2)
Abdominal Pain	42 (9.4)	7 (1.6)	0	28 (6.3)	5 (1.1)	0
Dyspepsia	31 (6.9)	0	0	29 (6.5)	0	0
Stomatitis	26 (5.8)	0	0	13 (2.9)	0	0
Dry Mouth	18 (4.0)	0	0	13 (2.9)	0	0
Abdominal Pain Upper	14 (3.1)	1 (0.2)	0	4 (0.9)	0	0
Dysphagia	7 (1.6)	2 (0.4)	0	23 (5.2)	5 (1.1)	0

Table 2: Treatment-emergent Adverse Events Reported by > 2% of Patients Receiving Romidepsin Monotherapy, by Indication and MedDRA SOC and Preferred Term, for All Adverse Events and Grade 3 and Grade 4 Adverse Events (N = 891) (Continued)

MedDRA SOC/Preferred Term	Indication					
	Hematologic Malignancies (N = 447)			Solid Tumors (N = 444)		
	All AEs n (%)	Grade 3 AEs n (%)	Grade 4 AEs n (%)	All AEs n (%)	Grade 3 AEs n (%)	Grade 4 AEs n (%)
**General Disorders and Administration Site Conditions**	**339 (75.8)**	**71 (15.9)**	**6 (1.3)**	**245 (55.2)**	**40 (9.0)**	**1 (0.2)**
Fatigue	237 (53.0)	43 (9.6)	2 (0.4)	210 (47.3)	29 (6.5)	0
Pyrexia	114 (25.5)	17 (3.8)	2 (0.4)	34 (7.7)	2 (0.5)	0
Chills	35 (7.8)	2 (0.4)	0	11 (2.5)	0	0
Asthenia	35 (7.8)	5 (1.1)	0	6 (1.4)	0	0
Oedema Peripheral	31 (6.9)	2 (0.4)	0	25 (5.6)	2 (0.5)	0
Pain	27 (6.0)	6 (1.3)	0	18 (4.1)	5 (1.1)	0
Oedema	27 (6.0)	3 (0.7)	2 (0.4)	6 (1.4)	0	0
Chest Pain	16 (3.6)	4 (0.9)	0	9 (2.0)	1 (0.2)	0
Injection Site Reaction	12 (2.7)	0	0	4 (0.9)	1 (0.2)	0
Non-Cardiac Chest Pain	11 (2.5)	1 (0.2)	0	6 (1.4)	1 (0.2)	0
Death	2 (0.4)	0	0	28 (6.3)	0	1 (0.2)
**Hepatobiliary Disorders**	**37 (8.3)**	**5 (1.1)**	**3 (0.7)**	**2 (0.5)**	**0**	**0**
Hyperbilirubinaemia	32 (7.2)	4 (0.9)	3 (0.7)	1 (0.2)	0	0
**Infections and Infestations**	**224 (50.1)**	**88 (19.7)**	**14 (3.1)**	**52 (11.7)**	**15 (3.4)**	**2 (0.5)**
Infection	39 (8.7)	17 (3.8)	3 (0.7)	6 (1.4)	3 (0.7)	1 (0.2)
Upper Respiratory Tract Infection	28 (6.3)	3 (0.7)	0	5 (1.1)	0	0
Urinary Tract Infection	24 (5.4)	4 (0.9)	0	10 (2.3)	0	0
Skin Infection	16 (3.6)	5 (1.1)	0	2 (0.5)	0	0
Sepsis	15 (3.4)	7 (1.6)	6 (1.3)	0	0	0
Neutropenic Infection	15 (3.4)	12 (2.7)	2 (0.4)	0	0	0
Catheter Related Infection	13 (2.9)	12 (2.7)	0	5 (1.1)	1 (0.2)	0
Staphylococcal Infection	13 (2.9)	4 (0.9)	0	1 (0.2)	0	0
Pneumonia	11 (2.5)	6 (1.3)	0	2 (0.5)	2 (0.5)	0
Nasopharyngitis	13 (2.9)	0	0	2 (0.5)	0	0
Cellulitis	11 (2.5)	6 (1.3)	0	1 (0.2)	0	0
Oral Candidiasis	11 (2.5)	2 (0.4)	0	1 (0.2)	0	0
Pharyngitis	10 (2.2)	0	0	1 (0.2)	0	0

Table 3: Treatment-emergent Adverse Events Reported by > 2% of Patients Receiving Romidepsin Monotherapy, by Indication and MedDRA SOC and Preferred Term, for All Adverse Events and Grade 3 and Grade 4 Adverse Events (N = 891) (Continued)

MedDRA SOC/Preferred Term	Indication					
	Hematologic Malignancies (N = 447)			Solid Tumors (N = 444)		
	All AEs n (%)	Grade 3 AEs n (%)	Grade 4 AEs n (%)	All AEs n (%)	Grade 3 AEs n (%)	Grade 4 AEs n (%)
**Investigations**	**232 (51.9)**	**58 (13.0)**	**30 (6.7)**	**220 (49.5)**	**74 (16.7)**	**9 (2.0)**
Electrocardiogram T-Wave Amplitude Decreased	86 (19.2)	0	0	0	0	0
Aspartate Aminotransferase Increased	66 (14.8)	7 (1.6)	1 (0.2)	50 (11.3)	3 (0.7)	0
Alanine Aminotransferase Increased	58 (13.0)	11 (2.5)	1 (0.2)	53 (11.9)	2 (0.5)	0
Platelet Count Decreased	47 (10.5)	16 (3.6)	15 (3.4)	125 (28.2)	12 (2.7)	1 (0.2)
Weight Decreased	42 (9.4)	0	0	40 (9.0)	0	0
Electrocardiogram QT Prolonged	37 (8.3)	4 (0.9)	0	21 (4.7)	6 (1.4)	0
Neutrophil Count Decreased	36 (8.1)	16 (3.6)	16 (3.6)	61 (13.7)	29 (6.5)	5 (1.1)
Blood Creatinine Increased	35 (7.8)	0	0	29 (6.5)	3 (0.7)	0
Blood Alkaline Phosphatase Increased	25 (5.6)	1 (0.2)	0	36 (8.1)	3 (0.7)	0
Electrocardiogram ST Segment	20 (4.5)	0	0	1 (0.2)	0	0
Blood Creatine Phosphokinase Increased	18 (4.0)	1 (0.2)	1 (0.2)	5 (1.1)	0	0
Lymphocyte Count Decreased	13 (2.9)	9 (2.0)	1 (0.2)	57 (12.8)	30 (6.8)	3 (0.7)
Activated Partial Thromboplastin Time Prolonged	8 (1.8)	1 (0.2)	0	13 (2.9)	4 (0.9)	0
International Normalised Ratio Increased	3 (0.7)	0	0	13 (2.9)	3 (0.7)	0
Blood Bilirubin Increased	1 (0.2)	0	0	28 (6.3)	3 (0.7)	0
Electrocardiogram Abnormal	1 (0.2)	1 (0.2)	0	14 (3.2)	0	0

- <u>Combination with other agents</u>

As of 31 December 2011, a total of 125 patients received romidepsin in combination with another chemotherapeutic agent (i.e., gemcitabine, flavopiridol, decitabine, rituximab), regardless of indication. Among these 90 patients, the most frequently reported adverse events overall were similar to those reported for romidepsin monotherapy and included nausea, thrombocytopenia, vomiting NOS, anemia, and fatigue.

3.4.2 *Special Risk Considerations for Romidepsin*

Cardiac Risks: QTc prolongation as well as several morphological changes in ECGs (including T wave and ST segment changes) have been reported in clinical studies. Many of the ECG morphologic abnormalities were also observed at baseline. These ECG changes were transient and

were not associated with functional cardiovascular changes or with symptoms. The clinical significance of these changes is unknown.
The potential effect of romidepsin on the heart-rate corrected QTc/QTcF was evaluated in 26 subjects with advanced malignancies given romidepsin at doses of 14 mg/m2 as a 4-hour intravenous infusion, and at doses of 8, 10 or 12 mg/m2 as a 1–hour infusion. No concentration-dependent effect of romidepsin on the duration of the QTc interval was identified at Cmax values up to 2.5-fold higher on average than observed with the clinical dose regimen of 14 mg/m2 administered as a 4-hour infusion.

3.4.3 *Special risk considerations for combination Romidepsin CHOP*

A phase I study of different doses of romidepsin (on day 1 and 8 of 21 day cycles for 8 cycles) plus CHOP was conducted by LYSARC. The tested romidepsin doses were 8 mg/m2, 10 mg/m2, and 12 mg/m2. A total of 18 patients were included in this dose escalating study, 3 at 8 mg/m2, 9 at 10 mg/m2, and 6 at 12 mg/m2. The most frequent AE was thrombocytopenia, particularly during cycle 1 (5 grade 3/4 events) and neutropenia (12 grade 3/4 events) without severe infection. The recommended dose for the expansion phase is 12mg/m² administered at day 1 and day 8 of each cycle.

Table 4: Treatment-emergent Serious Adverse Events Reported in Phase Ib study of romidepsin plus CHOP (May 25th, 2012) by MedDRA SOC (N = 18)

MedDRA SOC	Grade 1-2 AEs n	Grade 3 AEs n	Grade 4 AEs n
General disorders and administration site conditions	1	2	0
Gastrointestinal disorders	2	0	0
Vascular disorders	0	1	0
Respiratory, thoracic and mediastinal disorders	1	1	0
Cardiac disorders	1	0	0
Infections and infestations	0	1	0
Renal and urinary disorders	1	0	0
Investigations	0	0	1
Blood and lymphatic system disorders	0	2	0
Nervous system disorders	0	3	0

4 PATIENT SELECTION CRITERIA

Newly diagnosed patients with Peripheral T-cell lymphomas including: Peripheral T-cell lymphomas not otherwise specified (PTCL-NOS), Angioimmunoblastic T-cell lymphoma (AITL), ALK negative Anaplastic large-cell lymphoma (ALCL).

4.1 Inclusion criteria

1. age ≥18 e ≤ 65 years
2. Peripheral T-cell lymphomas at diagnosis including: PTCL-NOS, AITL, ALK negative ALCL
3. Stage II-IV
4. Written informed consent
5. No prior treatment for lymphoma
6. No Central Nervous System (CNS) disease (meningeal and/or brain involvement by lymphoma)
7. HIV negativity
8. Absence of active hepatitis C virus (HCV) infection
9. HBV negativity or patients with HBcAb +, HBsAg -, HBs Ab+/- with HBV-DNA negativity (in these patients Lamivudine prophylaxis is mandatory)
10. Levels of serum bilirubin, alkaline phosphatase and transaminases < 2 the upper normal limit, if not disease related
11. No psychiatric illness that precludes understanding concepts of the trial or signing informed consent
12. Ejection fraction > 50% and no myocardial stroke in the last year nor QT prolongation (QTc interval < 480 msec using the Fridericia formula)
13. Clearance of creatinine > 60 ml/min if not disease related
14. Spirometry Diffusion Capacity (DLCO) > 50%
15. Absence of active, uncontrolled infection
16. For males and females of child-bearing potential, agreement upon the use of effective contraceptive methods prior to study entry, for the duration of study participation and in the following 90 days after discontinuation of study treatment
17. Availability of histological material for central review and pathobiological studies.

4.2 Exclusion criteria

1. age <18 e > 65 years
2. Hystology other than: PTCL-NOS, AITL, ALKnegative ALCL
3. Stage I
4. Prior treatment for lymphoma
5. Positive serologic markers for human immunodeficiency virus (HIV)
6. Active hepatitis B virus (HBV) infection
7. Active hepatitis C virus (HCV) infection
8. Levels of serum bilirubin, alkaline phosphatase and transaminases > 2 the upper normal limit, if not disease related
9. Ejection fraction < 50% and myocardial stroke in the last year or QT prolongation (QTc interval > 480 msec using the Fridericia formula)
10. Clearance of creatinine < 60 ml/min if not disease related
11. Spirometry Diffusion Capacity (DLCO) < 50%

12. Pregnancy or lactation
13. Patient not agreeing to take adequate contraceptive measures during the study
14. Psychiatric disease that precludes understanding concepts of the trial or signing informed consent
15. Any active, uncontrolled infection
16. Prior history of malignancies other than PTCLs in the last five years (except for basal cell or squamous cell carcinoma of the skin or carcinoma *in situ* of the cervix or breast).

5 PHASE I PART OF THE STUDY

5.1 Objectives of the study

Primary objective

- To define the maximum tolerated dose (MTD) of Ro-CHOEP-21

Secondary objective

- To assess the feasibility of the Ro-CHOEP-21 treatment strategy combined with SCT

5.2 End-points

Primary endpoint

- Incidence of dose-limiting toxicity (DLT) of Ro-CHOEP-21, considering as maximum dose the one causing induction of any grade ≥ 3 non hematologic toxicity or a delay >15 days of planned cycle date observed during the first two cycles according to the definitions of NCI Common Terminology Criteria for Adverse Events (CTCAE), version 4.0 (2009)

Secondary endpoints

- Proportion of patients reaching SCT.
- Overall response rate (ORR, defined according to the Lugano Classification 2014 response criteria) of the combination of Ro-CHOEP-21.

5.3 Statistical design

The continual reassessment method (CRM) for dose-finding phase I study *(Zohar, 2001; O'Quigley and Zohar, 2006)* will be used as the dose allocation rule in the trial for groups of three patients at each dose. The design of this dose-finding phase clinical trial is chosen to assess the maximum tolerated dose (MTD) of romidepsin when administered in combination with CHOEP chemotherapy in the treatment of patients with T-cell lymphoma, candidate to stem cell transplant. The MTD is defined as the dose that achieves a dose-limiting toxicity (DLT) in 33% of patients. Four dose levels are tested, namely 8, 10, 12 and 14 mg/sqm. The CRM method is based on a mathematical modelling of dose–DLT relationship, iteratively updated using Bayes theorem along the trial, as follows. First, before trial onset, prior opinions about DLT probability at each dose level are elicited from expert clinicians on the basis of their personal experience and on literature. These initial guesses, which relied on the opinion of participating clinicians, were fixed at 0.15, 0.20, 0.25, and

0.30, respectively. The uncertainty in this dose–DLT relationship is incorporated into a prior. Then, the first three included patients are administered the third dose level (12 mg/sqm). After the enrollment of the first three patient, accrual continues, with grouped inclusions of three patients per dose level. Then, on the basis of observed responses (DLT or not), DLT probabilities of all dose levels are updated using Bayes theorem. The dose level associated with an updated DLT probability close to 33% is recommended to be administered to the next patient cohort. All this process is re-run until the fixed sample size (N=24) is reached, or in case of fulfilled stopping criteria measuring futility of trial continuation *(Zohar, 2003).*

5.3.1 *Study population*

Sample size: 21-24 patients (estimated 50% treated at the MTD)

5.3.2 *Study design and treatment*

The study consists of the following consecutive phases: A1) Induction phase and A2) stem cell mobilization and transplantation phase. Newly diagnosed patients will receive induction treatment with Romidepsin in combination with CHOEP-21 for 3 cycles, CR or PR patients will receive 3 additional courses, while not responders will be switched to an early salvage treatment and censored as a failure. To define the maximum tolerated dose (MTD), four dose levels of Romidepsin will be tested. The dose of Romidepsin will be modulated according to continual reassessment method. The starting dose for the first three patients will be 12 mg/ms (based on expert opinion). Stem cell mobilization will be with a DHAP course followed by G-CSF. At the end of induction, patients in CR/CRu will receive auto-SCT and patients in PR allo-SCT.

6 PHASE II PART OF THE STUDY

6.1 Objectives of the study

Primary objective

- To evaluate the efficacy in term of Progression Free Survival (PFS) of Ro-CHOEP-21

Secondary objectives

- To evaluate ORR and in particular CR rate achieved before and after SCT.
- To evaluate event free survival (EFS) and overall survival (OS)
- To evaluate the safety of treatment
- To evaluate the outcome of early allogeneic SCT in patients in PR at the end of induction phase
- To estimate the treatment-related mortality (TRM)
- To evaluate the incidence of acute and chronic GVHD in allografted patients
- To improve the knowledge on PTCL diagnosis, classification and biology.

Exploratory Objective

- Evaluation of response biomarkers (eg TET2 mutations)

6.2 Endpoints

Primary endpoint

- PFS on intention to treatment (ITT) evaluated at 18 months. PFS will be defined as the time between the date of enrolment and the date of disease progression, relapse or death from any cause.

Secondary endpoints

- ORR and CR (defined according to the Lugano Classification 2014 response criteria), after induction treatment and after SCT.
- Event free survival (EFS) defined as the time between the date of enrollment and the date of discontinuation of treatment for any reason
- Overall survival (OS) defined as the time between the date of enrolment and the date of death from any cause in the ITT population enrolled in the study
- PFS and OS in patients not responding to the first 3 courses of Ro-CHOEP-21
- Any grade III or higher toxicities, recorded and classified according to the definitions of NCI Common Terminology Criteria for Adverse Events (CTCAE), version 4.0 (2009)
- Evaluation during the interim analyses of any grade III or higher toxicities, recorded and classified according to the definitions of NCI Common Terminology Criteria for Adverse Events (CTCAE), version 4.0 (2009)
- Evaluation during all the pretransplant phase of any grade III or higher toxicities, recorded and classified according to the definitions of NCI Common Terminology Criteria for Adverse Events (CTCAE), version 4.0 (2009)
- Treatment-related mortality defined as any death that was not attributable to the lymphoma.
- Incidence of acute and chronic GVHD in allografted patients

Exploratory endpoint

- Evaluation of response biomarkers (eg TET2 mutations)

6.3 Statistical design

The sample size of this single arm, phase II trial, has been calculated using the PFS as the primary endpoint, according to the two-stage design proposed by Case and Morgan (2003), without interim pause in the enrolment. According to available evidence, the 1.5 year PFS of newly diagnosed PTCL patients treated with anthracycline based therapy (CHOP/CHOEP), followed by auto-SCT, is around 55% (null hypothesis, H0). With our experimental strategy, based on 6 courses of CHOEP-21 plus Romidepsin, followed by SCT in chemosensitive disease, we hypothesize to achieve an overall 1.5 year PFS of 70% (alternative hypothesis, H1).
To demonstrate an absolute improvement from 55% (literature data) to 70% of the 1.5 year PFS, with an alpha error of 0.05 (one tail), a beta error of 0.10, and assuming 3 years of constant accrual and at least 1.5 years of follow-up after the enrolment of the last patient, the required total sample size calculated to minimize the ETSL (expected total study length) is 110 (sample size calculated with the Sample Size Tables for Clinical Studies, 3rd edition, by Machin et al, 2009).
With this design the interim analysis will be performed when the first 75 patients have been enrolled. At this time the Kaplan-Meier 1.5 years PFS will be estimated, with its standard error, to calculate the Z interim test. To proceed with the enrolment, the threshold of the Z interim test for efficacy should be at least 0.650.

If this case, 35 further patients will be enrolled to reach the planned total sample size of 110 and the final analysis will be performed after the last enrolled patient has been followed for 18 months. To reject the null hypothesis the threshold of the Z final statistic must be greater than 1.522.

6.3.1 *Study population*

110 patients in total, including approximately 9-15 patients expected from the phase I study (treated at the MTD).

6.3.2 *Duration of the study*

Expected accrual time: 3 years after the Ethics Committee approval of all the centres and at least eighteen months of follow-up after the enrolment of the last patient.
Duration of treatment: 6-8 months
Duration of follow up: 5 years from the end of therapy or last patient enrolled.

7 STUDY TREATMENT

The study consists of the following consecutive phases: A1) Induction phase and A2) stem cell mobilization and transplantation phase. Newly diagnosed patients will receive induction treatment with Romidepsin in combination with CHOEP-21 for 3 cycles, CR or PR patients will receive 3 additional courses, while not responders will be switched to an early salvage treatment (according to each center guidelines) and censored as a failure. At the end of induction, patients in PR or CR/CRu will receive transplantation as consolidation treatment after the first 3 Ro-CHOEP courses.

A1) INDUCTION PHASE: according to the response achieved after 3 cycles of Ro-CHOEP-21 , patients in PR or CR will receive 3 additional Ro-CHOEP courses. Patients with less than PR or progressive disease will be treated with salvage treatments according to each institutional policy.

Ro-CHOEP-21

- Romidepsin* at the dose established by protocol iv (at the allocated dose of 8 or 10 or 12 or 14 mg/ms in phase I part, or at the MTD in phase II part) day +1 and +8
- Doxorubicin 50 mg/ms iv day +1,
- Vincristin 1.4 mg/ms (capped at 2.0 mg) iv day+1,
- Cyclophosphamide 750 mg/ms iv day +1,
- Etoposide 100mg/ms iv from day +1 to +3
- Prednisone 100 mg orally from days +1 to +5
- G-CSF sc from day +5 to ANC recovery or Pegfilgrastim 6 mg sc on day +4
- Courses repeated every 21 days

*Romidepsin should be administered first, before CHOEP, by a 4-hours infusion.

A2) STEM CELL MOBILIZATION AND TRANSPLANTATION PHASE: after Ro-CHOEP-21 for 6 courses, a response evaluation is performed. Patients in CR receive one course of DHAP followed by peripheral stem cell harvesting. High dose phase is BEAM or FEAM or CEAM followed by auto-SCT. Patients in PR will receive one course of DHAP followed by peripheral stem cell harvesting, but are candidate to allo-SCT. When a suitable donor is not available, PR patients

will receive auto-SCT: patients with progressive disease will be treated with salvage treatment according to each institutional policy.

DHAP

In-patient version

- Cisplatin 100 mg/ms iv day 1 in 24-hours infusion
- Cytarabine 2000 mg/ms in 3-hours infusion every 12 hours iv day 2
- Dexametasone 40 mg iv day 1-4
- G-CSF 5 µcg/kg/day sc starting from day +5 until peripheral blood stem cell harvest, when circulating CD34+ cells are >=20 per mcl

Out-patient version

- Cisplatin 100 mg/ms iv day 1 in 3-6-hours infusion
- Cytarabine 2000 mg/ms in 3-hours infusion iv day 2 and day 3
- Dexametasone 40 mg iv day 1-4
- G-CSF 5 µcg/kg/day sc starting from day +5 until peripheral blood stem cell harvest, when circulating CD34+ cells are >= 20 per microliter

CONDITIONING REGIMEN FOR AUTO-SCT: BEAM or FEAM or CEAM

- BCNU 300 mg/ms iv day -7 (BCNU can be replaced with Fotemustine 300 mg/ms or with CCNU 200 mg/ms)
- Cytarabine 200 mg/ms every 12 hours iv days -6, -5, -4, -3 (8 total doses)
- Etoposide 100 mg/ms every 12 hours iv days -6, -5, -4, -3 (8 total doses)
- Melphalan 140 mg/ms iv day -2
- Reinfusion of PBSC (CD34+ > 3 x10^6/Kg) day 0
- Day +3 G-CSF sc until neutrophil recovery

ALLO-SCT: DONOR MATCHING, MOBILIZATION AND HARVEST OF HEMATOPOIETIC CELLS

Patients are required to have an HLA-identical (A, B, C, DR, DQ loci) or one antigen mismatched (Class I) sibling donor, willing and capable of donating G-CSF-stimulated peripheral blood hematopoietic cells or bone marrow. Donor selection is based on molecular high-resolution typing (4 digits) of the HLA gene loci class I (HLA-A, B, and C) and class II (DRB1, DQB1). It is advisable to perform an exercise EKG testing in donors above 55 years of age or heavy smokers or suffering of hypertension or diabetes. Suitable sibling donors will receive lenogastrim or filgrastim 5 mcg/kg subcutaneously every 12 hours; on day +5 or +6, large volume leukapheresis will be performed. Target value of $CD34^+$ cells will be 5 x 106/kg of the recipient body weight (range, 4 to 8 x 106/kg). In case of a sibling donor unwilling or not suitable to G-CSF administration, a bone marrow harvest will be performed. We aim at a nucleated cell dose of at least 3 x 108/kg of recipients body weight. To achieve this, it is mandatory to aspirate between 20 and 30 ml of marrow blood per kilogram of donor’s body weight. Therefore a donation of 2 units of blood is requested from the donor in a month prior to harvest, to be used for auto-transfusions during the procedure. In

case, no class I and class II completely identical unrelated donor (10 out of 10 gene loci) can be identified, the degree of histocompatibility between patient and donor must fulfill with the minimal degree of matching established by the Italian Bone Marrow Donor Registry: HLA-A and HLA-B antigen histocompatibility and HLA-DRB1 allelic histocompatibility. For patients allografted from unrelated donor, the source of stem cells will be peripheral blood or bone marrow stem cells (3x10e8/kg total nucleated stem cells in case of bone marrow and > or = 4 – 8 x10e6/kg CD34+ in case of peripheral blood stem cells).

CONDITIONING REGIMEN FOR ALLO-SCT

Thiotepa total dose is 15mg/kg iv (10mg/kg if age >55ys or HCT comorbidity score >=2). Thiotepa 5 mg/kg every 12 hours iv for 2 or 3 doses (day –6 and/or -5); cyclophosphamide 30 mg/kg iv (days –4 and –3); fludarabine 30 mg/ m^2 iv (days –4 and –3), 4 hours post-cyclophosphamide administration; transplantation of 4 - 8 X 10^6 / kg CD 34+ cells on day 0 .

GVHD PROPHYLAXIS

GVHD prophylaxis consists of cyclosporin A 1 mg/kg/day, from day –6 to day –1, and then 2 mg/kg/day iv as a continuous infusion or orally in a twice-daily divided dose (total dose 4 mg/kg/day) if patients are able to take regularly oral feeding. Doses will be adjusted to maintain whole-blood steady-rate through levels at 200 to 300 ng/mL (using the monoclonal assay to assess cyclosporin blood levels), and modified as clinically indicated for nephrotoxicity. Methotrexate 10 mg/ms iv on day +1, methotrexate 8 mg/ms on days +3 and +6, followed 24 hour later by a single dose of leucovorin rescue at 25 mg/ms. In case of grade 3 renal or liver toxicity, or severe mucositis methotrexate will be omitted and mycophenolate 20 mg/kg/die will be started at day -1 for 30 days. Patients with a class I antigen mismatch (sibling donor) or with fully matched unrelated donor will receive Thymoglobuline starting with a low dose to decrease the infusion related symptoms (Thymoglobuline 0.5 mg/kg daily on day -4, Thymoglobuline 2 mg/kg on day –3 and Thymoglobuline 2.5 mg/kg –2). Premedication will include dexamethasone 8 mg, paracetamol 500 mg, anti-H1 and anti-H2 every 12 hrs. In case of mismatched unrelated donors the total dose of Thymoglobuline will be 7 mg/kg.
For patients in CR or PR, cyclosporin A will be administered at full dose through day +180 and, if GVHD will be absent, the dose will be tapered by 10% every 10 days thereafter. Patients with stable or progressive disease after transplant will rapidly taper cyclosporin (in 14 days) and then receive CD3+ lymphocytes if aGVHD or response has not occurred.

A1) **<u>Induction phase</u> (PHASE I)**		
Ro-CHOEP-21 x 3 cycles		
	Romidepsin (dose escalation)	*Starting dose: 12mg/ms* iv day +1 and +8<br>*Dose modification according to toxicity:*<br>• *14mg/ms day +1 and +8*<br>• *10mg/ms day +1 and +8*<br>• *8mg/ms day +1 and +8*
	CHOEP-21	• *Doxorubicin 50 mg/ms* iv *day +1,*<br>• *Vincristin 1.4 mg/ms (maximum 2.0 mg total dose)* iv *day+1,*<br>• *Cyclophosphamide 750 mg/ms* iv *day +1,*<br>• *Etoposide 100mg/ms* iv *from day +1 to +3*<br>• *Prednisone100 mg orally from days +1 to*

		+5
According to the response achieved after the first 3* Ro-CHOEP-21 *cycles:		
PR or CR	Ro-CHOEP-21 for 3 additional cycles followed by phase_A2	
SD or PD	Treatment failures, proceed to salvage according to each institutional policy	
A2) **<u>Stem cell mobilization and transplantation phase</u>**		
	Response evaluation and one DHAP course followed by peripheral stem cell harvesting	• *Dexamethasone 40mg* iv *day +1 +2 +3 +4*<br>• *Cisplatin 100mg/ms* iv *day +1*<br>• *Cytarabine 2gr/ms bid* iv *day +2(in-patient version) or Ara-C 2 gr/ms* iv *day +2 and day +3 (out-patient version)*<br>• *G-CSF 5 μcg/kg/day* sc *starting from day +5 until peripheral blood stem cell harvest*
According to response achieved after 6 Ro-CHOEP-21 cycles:		
CR	BEAM or FEAM or CEAM followed by auto-SCT	• *BCNU 300 mg/ms* iv *day -7 (or Fotemustine150 mg/ms* iv *day -7, -6 or 300mg/mq day -7; or CCNU 200 mg/ms iv day -7)*<br>• *Etoposide 200 mg/ms* iv *day -6,-5,-4, 3*<br>• *Cytarabine 200 mg/mq bid* iv *day -6,-5,-4, -3*<br>• *Melphalan140 mg/ms* iv *day-2*
PR	• **Allogeneic SCT** with HLA-identical (A, B, C, DR, DQ loci) or one antigen mismatched (class I) sibling donors. Donor selection is based on molecular high-resolution typing (4 digits) of the HLA gene loci class I (HLA-A, B, and C) and class II (DRB1, DQB1). In case, no class I and class II completely identical urelated donor (10 out of 10 gene loci) can be identified, the degree of histocompatibility between patient and donor must fulfill with the minimal degree of matching established by the Italian Bone Marrow Donor Registry: HLA-A and HLA-B antigen histocompatibility and HLA-DRB1 allelic histocompatibility.<br>• when a suitable donor is not available: BEAM or FEAM or CEAM followed by Auto-SCT	• *Thiotepa 15mg/kg (5mg/kg every 12 hours for 3 doses* iv *on day –6 and -5)*<br>• *or Thiotepa 10mg/kg(5mg/kg every 12 hours for 2 doses* iv *on day -5) if age >55yrs or Hematopoietic Cell Transplant-Comorbidity Index≥2)*<br>• *Cyclophosphamide 30mg/kg* iv *day -4, -3*<br>• *Fludarabine 30mg/mg* iv *day-4, -3*<br>• *GvHD prophylaxis: cyclosporine and short course methotrexate*
< PR	Treatment failures, proceed to salvage according to each institutional policy	

A1) **<u>Induction phase</u> (PHASE II)**		
Ro-CHOEP-21 x 3 cycles		
	Ro-CHOEP-21	• *Romidepsin dose according to phase I* iv *day +1 and +8*<br>• *Doxorubicin 50 mg/ms* iv *day +1,*<br>• *Vincristin 1.4 mg/ms (maximum 2.0 mg total dose)* iv *day+1,*

		• *Cyclophosphamide 750 mg/ms* iv *day +1,*<br>• *Etoposide 100mg/ms* iv *from day +1 to +3*<br>• *Prednisone100 mg orally from days +1 to +5*
According to the response achieved after the first 3* Ro-CHOEP-21 *cycles:		
PR or CR	Ro-CHOEP-21 for 3 additional cycles followed by phase_A2	
SD or PD	Treatment failures, proceed to salvage according to each institutional policy	
A2) **Stem cell mobilization and transplantation phase**		
	Response evaluation one DHAP course followed by peripheral stem cell harvesting	• *Dexamethasone 40mg* iv *day +1 +2 +3 +4*<br>• *Cisplatin 100mg/ms* iv *day +1*<br>• *Cytarabine 2gr/ms bid* iv *day +2(in-patient version) or Ara-C 2 gr/ms* iv *day +2 and day +3 (out-patient version)*<br>• *G-CSF 5 μcg/kg/day* sc *starting from day +5 until peripheral blood stem cell harvest*
According to response achieved after 6 Ro-CHOEP-21cycles:		
CR	BEAM or FEAM or CEAM followed by Auto-SCT	• *BCNU 300 mg/ms* iv *day -7 (or Fotemustine150 mg/ms* iv *day -7, -6 or 300mg/mq day -7; or CCNU 200 mg/ms iv day -7)*<br>• *Etoposide 200 mg/ms* iv *day -6,-5,-4, 3*<br>• *Cytarabine 200 mg/mq bid* iv *day -6,-5,-4, -3*<br>• *Melphalan140 mg/ms* iv *day-2*
PR	• **Allogeneic SCT** with HLA-identical (A, B, C, DR, DQ loci) or one antigen mismatched (class I) sibling donors. Donor selection is based on molecular high-resolution typing (4 digits) of the HLA gene loci class I (HLA-A, B, and C) and class II (DRB1, DQB1). In case, no class I and class II completely identical unrelated donor (10 out of 10 gene loci) can be identified, the degree of histocompatibility between patient and donor must fulfill with the minimal degree of matching established by the Italian Bone Marrow Donor Registry: HLA-A and HLA-B antigen histocompatibility and HLA-DRB1 allelic histocompatibility.<br>• when a suitable donor is not available: BEAM or FEAM or CEAM followed by Auto-SCT	• *Thiotepa 15mg/kg* iv *(5mg/kg every 12 hours for 3 doses on day –6 and -5)*<br>• *or Thiotepa 10mg/kg* iv *(5mg/kg every 12 hours for 2 doses on day -5) if >55 yrs or Hematopoietic Cell Transplant-Comorbidity Index≥2) day -5*<br>• *Cyclophosphamide 30mg/kg* iv *day -4, -3*<br>• *Fludarabine 30mg/mg* iv *day-4, -3*<br>• *GvHD prophylaxis: cyclosporine and short course methotrexate*
< PR	Treatment failures, proceed to salvage according to each institutional policy	

7.1 Registration of the patient and Romidepsin dose-allocation

A centrally online procedure to enroll patients and to dose allocation of Romidepsin is available on FIL website:

http://www.fililinf.it

At enrolment time, a numeric code will be assigned at each patient; the code will be published on "enrolled patients" section on website within 48 hours and will be sent by e-mail at the center. After the enrollment, it is not possible to change the therapeutic scheme attributed to the patient. If the patient, for personal or physician choice, refuse treatment, the patient is considered a failure.

8 PATHOLOGICAL REVIEW AND BIOLOGIC STUDIES

8.1 Pathological review

A central pathology review is planned at accrual for all patients enrolled into the trial.
An independent pathologist (Stefano Pileri, European Institute of Oncology) will review the lymph node/tumor biopsy slides for retrospective confirmation of the diagnosis of PTCL. Investigator centers are required to submit at minimum 10 unstained slides from the tumor/lymph node biopsy specimen taken at the time of the diagnosis or the paraffin block which will be returned as the histological revision has been done.

8.2 Biological studies

Tumor tissue samples will also be collected in this trial with the purpose of identifying biomarkers: TET2 mutations will be analyzed at the Istituto Nazionale Tumori, Milano and correlated with the clinical outcome.

Archival tumor tissue is mandatory when available, to participate in this explorative part of the study. It is preferred that archival tissue is provided as paraffin block. However if this is not possible, at least 10 unstained slides should be provided for each patient. If baseline BM have detectable tumor invasion, the shipment of this diagnostic sample is required. The biomarker analysis will be explored during the study. The diagnosis will be additionally refined using the Gene Expression Profiling (GEP)-based molecular classifiers (MCs) recently developed, able to accurately distinguish between PTCL NOS and either AITL or ALK^{-}ALCL.(Piccaluga et al JCO2013).
The collection of post-treatment tumor sample at disease progression is encouraged to investigate the potential mechanisms of resistance of Ro-CHOEP-21 in PTCL patients. If the patient consents and it is clinically feasible, it is encouraged to collect a fresh tumor biopsy at tumor progression for the biological study. This sample will be analyzed by using a combination of genomic and proteomic technology to identify the driving genetic mechanisms in chemorefractory PTCL.

The search for potential relevant biomarkers for Ro-CHOEP-21 effect, disease and/or safety could be extended from what reported above depending on clinical outcome, reagent and sample availability.

8.3 Blood for germline DNA

In patients providing tumor biopsy at study entry a separate whole peripheral blood sample (PB) will be obtained. This sample will be collected for comparing tumor-specific gene alterations in the DNA from tumor biopsies with the DNA from normal-non-tumor cells.

8.4 Operative considerations for sample shipment for the biological studies

Archival tumor specimens as paraffin block or slides, PB and BM blood samples (if invaded), will be collected at study entry and shipped to Istituto Nazionale Tumori, Milano using the provided courier.

- 9 ml of Peripheral Blood collected in 3 tubes for automated complete blood count (CBC) (eg: BD Vacuatainer K2EDTA spray coated tubes, product no 368856 or similar);

-10 ml Bone Marrow aspirate will be collected in a polypropylene tube (eg 15ml Falcon conical tubes product no 352097) containing 5000U Heparin (1 ml).

Samples must be shipped to the following address:

Dr.ssa Cristiana Carniti
Laboratorio di Ematologia – Trapianto di Midollo Osseo Allogenico
Fondazione IRCCS, Istituto Nazionale dei Tumori
via Venezian, 1 20133 Milano, Italy

8.5 Ethical Aspects of Biological studies

Eligible patients will be asked to give a signed informed consent to take part in the study. In order to maintain patients privacy, in all data records, study reports and communications, the patients will be identified by his initials and his assigned unique patient number (UPN).
The table connecting all the UPNs to the patients' information will be kept in the database of the S.C. Ematologia- Trapianto di Midollo Osseo Allogenico, Fondazione IRCCS-Istituto Nazionale dei Tumori, Milano. To enter the database a password is required so that all the data are kept confidential.
At least 10 unstained slides or paraffin blocks will be collected at study entry by Prof Pileri (European Institute of Oncology) for the pathological evaluation. In case paraffin blocks are provided, these will be returned to the investigator center of origin as the histological evaluation has been performed.
The biological samples sent to the Fondazione IRCCS, Istituto Nazionale dei Tumori will be kept in a locked -80C freezer located in a locked laboratory. The access to this premise is permitted only to person authorized by the lab manager (Dr. Cristiana Carniti).
The biological samples will only be used for the proposed study and the left over biological samples are at disposal of the patients.

9 STUDY TREATMENT AND CONCOMITANT TREATMENT

Dose Modification and Delay

9.1 Dose-adjustment for Romidepsin

If a Romidepsin dose due to toxicity or another reason is missed, then that dose is skipped and treatment continues with next planned dose.
No adjustment of dose is planned, according to design of the study.
Only in case of QTc > 500 msec or ventricular arrhythmia: VT (≥ 3 beats in a row), or new occurrence of > Grade 2 atrial fibrillation or flutter: do not administer the present dose, hold next dose of romidepsin and consult cardiologist prior to restart romidepsin. In case of ventricular fibrillation (VF) including Torsade de Pointes: stop romidepsin permanently.

9.2 Dose- adjustment for CHOEP

Before each course, blood count will be taken and, if at day 21 ANC <1000/mm^3 and/or platelets <75.000/mm^3, the whole regimen will be delayed by one week. If at day 28 the ANC is 1000-1500/mm^3 and/or PLT 75-100.000/mm^3 the dosage of each chemotherapeutic drug will be reduced at 75%. If blood count has not recovered one further delay-week is admitted.
If at day 35 ANC are still <1000//mm^3, and/or platelets < 75.000/mm^3, the patient will go off-study.

9.3 Dose -adjustment for DHAP

Before DHAP, blood count will be taken and, if at day 28 ANC <1000/mm^3 and/or platelets <75.000/mm^3, the whole regimen will be delayed by one week. If at day 35 the ANC is 1000-1500/mm^3 and/or PLT 75-100.000/mm^3 the dosage of each chemotherapeutic drug will be reduced at 75%. If blood count has not recovered one further delay-week is admitted.
If at day 42 ANC are still <1000//mm^3, and/or platelets < 75.000/mm^3, the patient will go off-study.

9.4 Recommended concomitant treatments

During treatment are recommended as concomitant therapy:

- Cotrimoxazole BACTRIM 3 tablets per week or Pentamidine aerosol every 15 days in patients with Bactrim allergy or in patients with G6PD deficiency throughout the treatment and consolidation phase
- In patients with Ab antiHBcAg +, Ab antiHBsAg +/- prophylaxis against hepatitis B reactivation with Lamivudine 100 mg/die from one week prior the start of the treatment to one year after the end of the treatment
- All concomitant medications for medical conditions other than lymphoma are permitted, as clinically indicated
- All supportive therapies considered a standard practice for the chemotherapy phase and auto- or allo-SCT phase are permitted. In particular those concerning anti-nausea treatment, CMV prophylaxis, anti-fungal prophylaxis or the treatment of infections.

9.5 Permitted concomitant therapy

The following medications and support therapies that may be used if needed during this study:

- Antiviral prophylaxis with acyclovir 800-1200 mg daily since the beginning of therapy is strongly recommended in patients at risk of herpes virus infection reactivation
- Additional prophylaxis with levofloxacine or ciprofloxacine and fluconazole will be administrated in case of neutropenia <1.0 x 109/l
- Plerixafor in addition to GSCF during mobilization is permitted
- Platelets and red blood cell transfusion are allowed, if needed. Packed red cells and platelets transfusions will be given with filtered and irradiated products in case of Hb < 8 g/dL or Plts < 10 x 109/L or higher in case of bleeding signs.
- Erytropoietin therapy is allowed according to ASH/ASCO guidelines.
- Bowel care is recommended to prevent constipation and should be administered per standard practice.
- Antiemetic agents.
- Allopurinole or rasburicase for tumor lysis syndrome prevention is allowed.

9.6 Prohibited concomitant therapy

The following medications and supportive therapies are prohibited at all times:

- Any antineoplastic agent other than those planned by the study program.
- Any experimental agent.

9.7 Drugs affecting Qtc

Use of concomitant medications that increase or possibly increase the risk to prolong the QTc interval and/or induce torsades de pointes, ventricular arrhythmia are not permitted.
For details related to the drug characteristics see

http://www.azcert.org/medical-pros/druglists/drug-lists.cfm .

9.8 Inhibitor or inducer of Cytochrome P450 3A4 Enzyme

Romidepsin is metabolized by CYP3A4. Although there are no formal drug interaction studies for Romidepsin, strong CYP3A4 inhibitors (e.g., ketoconazole, itraconazole, clarithromycin, atazanavir, indinavir, nefazodone, nelfinavir, ritonavir, saquinavir, telithromycin, voriconazole) may increase concentrations of Romidepsin.
Therefore, co-administration with strong CYP3A4 inhibitors should be avoided if possible.
Caution should be exercised with concomitant use of moderate CYP3A4 inhibitors.
Co-administration of potent CYP3A4 inducers (e.g., dexamethasone, carbamazepine, phenytoin, rifampin, rifabutin, rifapentine, phenobarbital) may decrease concentrations of Romidepsin and should be avoided if possible. Patients should also refrain from taking St. John’s Wort.

9.9 Inhibitor or drug transport systems

Romidepsin is a substrate of the efflux transporter P-glycoprotein (P-gp, ABCB1). If Romidepsin is administered with drugs that inhibit P-gp, increased concentrations of Romidepsin are likely, and caution should be exercised.

10 CLINICAL EVALUATION, LABORATORY TESTS AND FOLLOW-UP

10.1 Staging evaluation, baseline

Baseline assessment must be performed during 30 days before starting therapy.

- Complete medical history, ECOG performance status, physical examination, vital signs
- ECG with QTc calculation and echocardiogram or MUGA scan for LVEF evaluation
- Spirometry DLCO
- Complete blood count, hematology workup and biochemistry
- HBsAg, HBcAb, HCV and HIV serology
- Lymph-node or tissue biopsy for histological diagnosis and shipment of paraffin block for centrally pathology review and for biological studies
- Aspirate and bone marrow biopsy
- CT scan neck, chest, abdomen and pelvis
- Total body PET scan
- Pregnancy test (if applicable)
- Lumbar puncture
- Written informed consent
- If clinically indicated: neurological visit, RMN brain/spine, GI endoscopy, ORL visit
- HLA typing (not mandatory)

10.2 Evaluation at each Ro-CHOEP courses

- Blood count and complete workup with biochemistry, physical examination, vital signs and hematological and extrahaematological toxicity evaluation the day before or day 1 and day 8 of therapy and between two cycles and during aplasia phase and/or till granulocytes and platelets recovery
- Electrocardiogram will be performed just before romidepsin infusion (after administration of antiemetic premedication if possible) at day 1 of each cycle for measurement of corrected QT interval according to the Fridericia formula and in case of cardiac event or clinical signs compatible with heart rhythm disorder and in case of biological abnormalityes.

10.3 Intermediate response evaluation

The evaluation of intermediate response will be assessed after 3 courses of Ro-CHOEP-21.

- ECOG performance status, physical examination, vital signs
- Blood count and complete workup with biochemistry
- Aspirate and bone marrow biopsy (if positive at baseline)
- CT scan neck, chest, abdomen and pelvis
- Total body PET scan (not mandatory)
- HLA typing and start donor search mandatory for patients in PR

Responsive patients (in partial or complete response) after three cycles of therapy, will continue the trial and will be treated with three further courses of Ro-CHOEP as planned.

10.4 Post-Induction evaluation

The evaluation post induction will be assessed after six courses of Ro-CHOEP.

- ECOG performance status, physical examination, vital signs

- Blood count and complete workup with biochemistry
- Aspirate and bone marrow biopsy (if positive at baseline)
- CT scan neck, chest, abdomen and pelvis
- Total body PET scan

Patients in CR will receive one course of DHAP followed by peripheral stem cells harvesting and BEAM or FEAM or CEAM followed by autologous stem cell transplant; patients in PR will receive one course of DHAP followed by peripheral stem cells harvesting and allo-SCT; patients in PR when a suitable donor is not available, will receive one course of DHAP + PBSC harvest followed by BEAM or FEAM or CEAM followed by autologous stem cell transplant; patients in SD or progressive disease will receive salvage treatment according to each institutional policy outside the protocol. In patients in PR when a suitable donor is available, DHAP plus PBSC harvest can be avoided.

10.5 Post –SCT evaluation

Final evaluation will be performed one-two months after the end of SCT.

- ECOG performance status, physical examination, vital signs
- Blood count and complete workup with biochemistry
- Aspirate and bone marrow biopsy (if positive at baseline)
- CT scan neck, chest, abdomen and pelvis
- Total body PET scan

Complete response, Partial response or no response will defined according to Lugano 2014 response criteria.

10.6 Follow-up

The total duration of follow-up is 5 years with the following plan: every 3 months during the first year after chemotherapy and then every 6 months up to 3 years after chemotherapy and then annually for a further 2 years. At all these steps will be evaluated:

- ECOG performance status, physical examination, vital signs
- Blood count and complete workup with biochemistry
- CT scan neck, chest, abdomen and pelvis
- Total body PET scan when clinically indicated

11 FORMS AND PROCEDURES FOR COLLECTING DATA AND DATA MANAGING

Data will be collected online by electronic-crf; site

http://www.filinf.it

CRF is the primary data collection instruments for the study. All data requested on the CRF must be recorded, and any missing data must be explained. If a space is left blank because the procedure

was not done or the question was not asked, “N/D” must be noted. If the item is not applicable to the individual case “N/A” must be noted.
In clinical trials the CRF must be dated and signed by the responsible investigator or one of his/her authorized staff members.

12 ADVERSE EVENTS, SERIOUS ADVERSE EVENTS

Timely, accurate, and complete reporting and analysis of safety information from clinical studies are crucial for the protection of subjects and are mandated by regulatory agencies worldwide.

Definitions

Adverse Event Definitions and Classifications

12.1 Adverse Event

An adverse event is any untoward medical occurrence in a clinical study subject administered a pharmaceutical product. An adverse event does not necessarily have a causal relationship with the treatment. An adverse event can therefore be any unfavorable and unintended sign (including an abnormal finding), symptom, or disease temporally associated with the use of a medicinal (investigational or non-investigational) product, whether or not related to the medicinal (investigational or non-investigational) product. (Definition per International Conference on Harmonization [ICH])

This includes any occurrence that is new in onset or aggravated in severity or frequency from the baseline condition, or abnormal results of diagnostic procedures, including laboratory test abnormalities.

The Adverse Events collection for each subject will start with the signing of informed consent form.

12.2 Serious Adverse Event

A serious adverse event as defined by ICH is any untoward medical occurrence that at any dose meets any of the following conditions:

- results in death
- is life-threatening
  (the subject was at risk of death at the time of the event. It does not refer to an event that hypothetically might have caused death if it were more severe.)
- requires inpatient hospitalization or prolongation of existing hospitalization
- results in persistent or significant disability/incapacity, or is a congenital anomaly/birth defect
- other medically important condition: would be any important medical or clinical event that may not be immediately life-threatening or result in a fatality or hospitalization but that may jeopardize the patient or require intervention to prevent another outcome e.g significant/persistent disability, life-threatening reaction congenital anomaly. Examples of such potentially serious events (according to medical judgment) include a suspected transmission of infections agent by a medicinal product, allergic bronchospasm, blood dyscrasias or convulsion, development of drug dependency or drug abuse, cancer. Whether

the event should meet one of these requirements, please report into the category “Other Medically Important Condition” on the SAE form .

Note: Medical and scientific judgment should be exercised in deciding whether expedited reporting is also appropriate in situations other than those listed above. Any adverse event is considered a serious adverse event if it is associated with clinical signs or symptoms judged by the investigator to have a significant clinical impact.

12.3 Unlisted (Unexpected) Adverse Event

An unlisted adverse event, the nature or severity of which is not consistent with the applicable product information. For an investigational product, the expectedness of an adverse event will be determined by whether or not it is listed in the Investigator's Brochure. For a comparator product with a marketing authorization, the expectedness of an adverse event will be determined by whether or not it is listed in the summary of product characteristics (SmPC).

12.4 Associated with the Use of the Drug

An adverse event is considered associated with the use of the drug if the attribution is possible, probable, or very likely by the definitions listed in Section 12.6

12.5 Product Quality Complaint

A product quality complaint (PQC) is defined as a complaint specific to the product itself, its supporting devices or packaging, as opposed to its effect on the patient. Examples include damaged or missing tablets; wrong strength or color of tablets; damaged packaging; a label that cannot be read; a liquid that should be clear but is cloudy or contains unexpected particles; a bent needle; a broken syringe; a missing patient information leaflet, or the identification of a potentially counterfeit medicine. Patients will be instructed to return empty blister or unused capsules. Unused or returned study drug will be destroyed locally in compliance with local pharmacy destruction procedures and drug disposition must be appropriately documented in the study file. Furthermore, the documentation reporting the drug destruction have to be sent to local Medical Affairs Dept. in Celgene srl. The local pharmacy is responsible for the drug destruction, and Celgene srl is not involved in any related activity. If any study drug is lost or damaged, its disposition should be documented in the source documents.

12.6 Attribution Definitions

12.6.1 *Intensity (Severity) Reporting and Attribution*

For both serious and non-serious adverse events, the investigator must determine both the intensity of the event and the relationship of the event to study drug administration.

Intensity for each adverse event will be determined by using Version 4.0 of the National Cancer Institute Common Toxicity Criteria (NCI CTC) as a guideline (homepage http://ctep.info.nih.gov), wherever possible. The criteria will be provided to the investigator as a separate document. In those cases where the NCI CTC do not apply, intensity should be defined according to the following criteria:

- Mild: Awareness of sign or symptom, but easily tolerated, causing minimal discomfort and not interfering with everyday activities; no medical intervention/therapy is required.
- Moderate Discomfort: Enough to cause mild to moderate interference with normal daily activities, some assistance may be needed, no minimal medical intervention/therapy is required.
- Severe: Extreme distress causing significant impairment of functioning or incapacitation and inability to perform normal daily activities. Some assistance is usually required; medically intervention/therapy is required and hospitalization may be required
- Life Threatening: Extreme limitation in activity. Risk of death from the reaction as it occurred.

The investigator should use clinical judgment in assessing the intensity of events not directly experienced by the subject (eg, laboratory abnormalities).

Relationship to study drug administration will be determined as follows:

- Not related
  An adverse event which is not related to the use of the drug.
- Unlikely/Doubtful
  An adverse event for which an alternative explanation is more likely, e.g., concomitant drug(s), concomitant disease(s), or the relationship in time suggests that a causal relationship is unlikely.
- Possible
  An adverse event which might be due to the use of the drug. An alternative explanation, e.g., concomitant drug(s), concomitant disease(s), is inconclusive. The relationship in time is reasonable; therefore, the causal relationship cannot be excluded.
- Probable
  An adverse event which might be due to the use of the drug. The relationship in time is suggestive (e.g., confirmed by dechallenge). An alternative explanation is less likely, e.g., concomitant drug(s), concomitant disease(s).
- Definite/Very Likely

  An adverse event which is listed as a possible adverse reaction and cannot be reasonably explained by an alternative explanation, e.g., concomitant drug(s), concomitant disease(s). The relationship in time is very suggestive (e.g., it is confirmed by dechallenge and rechallenge).

12.7 Reporting Procedures

All Adverse Events

All adverse events will be registered in CRF from the time a signed and dated informed consent form is obtained until 30 days after the administration of the last dose of study drug. Those meeting the definition of serious adverse events must be reported using the Serious Adverse Event Form.

Serious Adverse events occurring after 30 days should be reported if considered at least possibly related to the investigational medicinal product by the investigator.

Clinically relevant changes in laboratory values must be recorded in the adverse event section of the CRF. For example, laboratory abnormalities leading to an action regarding the study drug (dose change, temporary stop, delay of the start of a cycle or permanent stop) or the start of concomitant therapy should be reported. For each laboratory abnormality reported as an adverse event, the following laboratory values should be reported in the laboratory section of the CRF: the value indicative of the onset of each toxicity grade, the most abnormal value observed during the adverse event, and the value supporting recovery to Grade 0 or 1 or to baseline condition.

All adverse events, regardless of seriousness, severity, or presumed relationship to study therapy, must be recorded using medical terminology in the source document and the CRF. Whenever possible, diagnoses should be given when signs and symptoms are due to a common etiology (e.g., cough, runny nose, sneezing, sore throat, and head congestion should be reported as "upper respiratory infection"). Investigators must record in the CRF their opinion concerning the relationship of the adverse event to study therapy. All measures required for adverse event management must be recorded in the source document and reported according to Sponsor-Investigator instructions.

The Sponsor-Investigator assumes responsibility for appropriate reporting of adverse events to the regulatory authorities. The Sponsor-Investigators will also report to the Investigator, Independent Ethics Committee/Institutional Review Board (IEC/IRB) and to the Italian Drug Agency (AIFA) all serious adverse events of this study that are unlisted and associated with the use of the drug.

Subjects must be provided with a "study card" indicating the name of the investigational product, the study number, the investigator's name, a 24-hour emergency contact number, and, if applicable, excluded concomitant medications.

Pregnancies

While pregnancy, in itself, is not an adverse event, any subject pregnancy or pregnancies in partners of male subjects included in the study must be submitted by investigational staff to the Sponsor-Investigator within 24 hours of their knowledge of the event using the pregnancy notification form.

Abnormal pregnancy outcomes are considered serious adverse events and must be reported using the Serious Adverse Event Form. Any subject who becomes pregnant during the study must be promptly withdrawn from the study.

Because the study drug may have an effect on sperm, or if the effect is unknown, pregnancies in partners of male subjects included in the study will be reported by the investigational staff within 24 hours of their knowledge of the event using the pregnancy notification form.

Follow-up information regarding the outcome of the pregnancy and any postnatal sequelae in the infant will be required.

Serious Adverse Events and/or Pregnancies and/or Product Quality Complaint

All SAEs (and/or Pregnancies and/or PQC) occurring during clinical studies must be reported to the appropriated Sponsor-Investigator's contact person by investigational staff within 24 hours of their knowledge of the event.

Information regarding SAEs (and/or Pregnancies and/or PQC) will be transmitted to the Sponsor-Investigator using the Serious Adverse Event Form (and/or the Product Quality Complaints Form),

which must be signed by a member of the investigational staff. It is preferable that serious adverse events be reported via fax. Subsequent to a telephone report of a serious adverse event (and/or a Pregnancy and/or a PQC), a Serious Adverse Event Form (and/or a PQC form) must be completed by the investigational staff and transmitted to the Sponsor-Investigator within 1 working day.

The SAE(s) and/or Pregnancy report(s) and/or PQC must be sent to the Sponsor-Investigator Pharmacovigilance Contact Person to the following fax number:

Sponsor contact:

Dr. Alessandro Levis

Address: c/o S.C. Ematologia Azienda Ospedaliera Santi Antonio e Biagio e Cesare Arrigo - Alessandria

Phone no.: +39-0131-206071-206066

Fax no.: +39-0131-263455

All serious adverse events that have not resolved by the end of the study, or that have not resolved upon discontinuation of the subject's participation in the study, must be followed until any of the following occurs:

- The event resolves
- The event stabilizes
- The event returns to baseline, if a baseline value is available
- The event can be attributed to agents other than the study drug or to factors unrelated to study conduct
- It becomes unlikely that any additional information can be obtained (subject or health care practitioner refusal to provide additional information, lost to follow up after demonstration of due diligence with follow-up efforts).

The cause of death of a subject in a clinical study, whether or not the event is expected or associated with the investigational agent, is considered a serious adverse event. Suspected transmission of an infectious agent by a medicinal product should be reported as a serious adverse event. Any event requiring hospitalization (or prolongation of hospitalization) that occurs during the course of a subject's participation in a clinical study must be reported as a serious adverse event, except hospitalizations for:

- social reasons in absence of an adverse event
- surgery or procedure planned before entry into the study (must be documented in the CRF)
- study drug administration
- study related procedures defined in the protocol.

13 ETHICAL CONSIDERATIONS

13.1 Patient protection

The responsible investigator will ensure that this study is conducted in agreement with either the Declaration of Helsinki (Tokyo, Venice, Hong Kong and Somerset West amendments) or the laws and regulations of the country, whichever provides the greatest protection of the patient.

The protocol has been written, and the study will be conducted according to the ICH Guideline for Good Clinical Practice

The protocol and its annexes are subject to review and approval by the competent Independent Ethics Committee(s) ("IEC").

14 SUBJECT IDENTIFICATION – PERSONAL DATA PROTECTION

All records identifying the subject must be kept confidential and, to the extent permitted by the applicable laws and/or regulations, not be made publicly available. The name of the patient will not be asked for nor recorded at the Data Center. A sequential identification number will be automatically attributed to each patient registered in the study. This number will identify the patient and must be included on all case report forms. In order to avoid identification errors, patient initials and date of birth will also be reported on the case report forms.

Any and all patient information or documentation pertaining to a clinical trial, to the extent permitting, through a "key" kept anywhere, regardless of whether such key is supplied along with the information or documentation or not, must be considered as containing sensitive personal data of the patient, and is therefore subjected to the provisions of applicable data protection ("privacy") regulations. Breach of such regulations may result in administrative or even criminal sanctions.

Particularly, an information sheet prepared according to such regulations and a form to evidence the consent of patients to the processing of such data must therefore accompany the informed consent administered to the patient (see paragraph 14.3 below). Such information must (i) identify the roles of the holder ("titolare") and processor ("responsabile", appointed by the holder) of the patient personal data (also if not directly identifying the patient), as well as the purposes of the personal data collection and processing (medical treatment and related/unrelated scientific research), (ii) adequately describe the flows of communication involving them, particularly if third parties should become involved, and (iii) seek the patient's prior and specific consent to such processing.

Patient information or documentation may be considered "anonymous", and as such not subject to privacy regulations, only when no key whatsoever, permitting the identification of the patient, is any longer available.

Particular attention should therefore be paid (and information/consent materials adapted accordingly) whenever patient data are supplied to third parties and may be autonomously processed, or biological samples/materials are taken and kept for future research purposes, associated or not with the pathology considered in the study.

A copy of Informed consent should be attached to this Protocol Template.

14.1 Informed consent

All patients will be informed of the aims of the study, the possible adverse events, the procedures and possible hazards to which he/she will be exposed, and the mechanism of treatment allocation. They will be informed as to the strict confidentiality of their patient data, but that their medical records may be reviewed for study purposes by authorized individuals other than their treating physician. An example of a patient informed consent statement is given as an appendix to this protocol.

It will be emphasized that the participation is voluntary and that the patient is allowed to refuse further participation in the protocol whenever he/she wants. This will not prejudice the patient's subsequent care. Documented informed consent must be obtained for all patients included in the study before they are registered or randomized at the Data Center. This must be done in accordance with the national and local regulatory requirements.

For European Union member states, the informed consent procedure must conform to the ICH guidelines on Good Clinical Practice. This implies that "the written informed consent form should be signed and personally dated by the patient or by the patient's legally acceptable representative".

A copy of Informed consent should be attached to this Protocol Template.

15 CONFLICT OF INTEREST

Any investigator and/or research staff member who has a conflict of interest with this study (such as patent ownership, royalties, or financial gain greater than the minimum allowable by their institution) must fully disclose the nature of the conflict of interest.

16 DATA OWNERSHIP

According to the Good Clinical Practice the sponsor of a study is the owner of the data resulting therefrom. All centers and investigators participating in the study should be made aware of such circumstance and invited not to disseminate information or data without the Institution's prior express consent.

17 PUBLICATION POLICY

This section should be adapted to the statutes of the cooperative group.

After completion of the study, the project coordinator will prepare a draft manuscript containing final results of the study on the basis of the statistical analysis. The manuscript will be derived to the co-authors for comments and after revision will be sent to a major scientific journal.

All publications, abstracts, presentations, manuscripts and slides including data from the present study will be submitted to and reviewed by the Study Coordinator for coordination and homogeneity purposes: specific advance periods for submission and review may be specified in the protocol. The timing of publications (in the event several Centers should be participating in the Study) may be coordinated, and publication delayed if patentable inventions should be involved (for the time required in order to file the relevant patent applications); otherwise, according to the MoH's Decree of May 12, 2006, investigators cannot be precluded from or limited in publishing the

results of their studies (IECs must verify that no excessive restriction is contained in the protocols submitted to their review and approval).

18 STUDY INSURANCE

The Investigator-sponsor of the Study must ensure that adequate insurance coverage is available to the patients, in accordance with Section 5.8 of the ICH Guidelines of Good Clinical Practice and with the Italian DM July 14, 2009. Such coverage must extend to all damages deriving from the study, to the exclusion of those attributable to willful misconduct or negligence of the institution or investigator. A copy, or excerpt, or insurer's certificate, attesting the existence and amount of such coverage at least for the duration of the study must be supplied as part of the study documentation to the review and approval of the IEC.

19 REFERENCES

1. Vose J, Armitage J, Weisenburger D. International peripheral T-cell and natural killer/T-cell lymphoma study: pathology findings and clinical outcomes. *J Clin Oncol* 26(25), 4124-4130 (2008).
2. Mounier N, Simon D, Haioun C, Gaulard P, Gisselbrecht C. Impact of high-dose chemotherapy on peripheral T-cell lymphomas. *J Clin Oncol* 20(5), 1426-1427 (2002).
3. Simon A, Peoch M, Casassus P *et al.* Upfront VIP-reinforced-ABVD (VIP-rABVD) is not superior to CHOP/21 in newly diagnosed peripheral T cell lymphoma. Results of the randomized phase III trial GOELAMS-LTP95. *Br J Haematol* 151(2), 159-166 (2010).
4. Gallamini A, Zaja F, Patti C *et al.* Alemtuzumab (Campath-1H) and CHOP chemotherapy as first-line treatment of peripheral T-cell lymphoma: results of a GITIL (Gruppo Italiano Terapie Innovative nei Linfomi) prospective multicenter trial. *Blood* 110(7), 2316-2323 (2007).
5. Schmitz N, Trumper L, Ziepert M *et al.* Treatment and prognosis of mature T-cell and NK-cell lymphoma: an analysis of patients with T-cell lymphoma treated in studies of the German High-Grade Non-Hodgkin Lymphoma Study Group. *Blood* 116(18), 3418-3425 (2010).
6. D'amore F, Relander T, Lauritzsen Gf *et al.* Up-front autologous stem-cell transplantation in peripheral T-cell lymphoma: NLG-T-01. *J Clin Oncol* 30(25), 3093-3099 (2012).
7. Dhedin N, Giraudier S, Gaulard P *et al.* Allogeneic bone marrow transplantation in aggressive non-Hodgkin's lymphoma (excluding Burkitt and lymphoblastic lymphoma): a series of 73 patients from the SFGM database. Societ Francaise de Greffe de Moelle. *Br J Haematol* 107(1), 154-161 (1999).

8. Kim Sw, Tanimoto Te, Hirabayashi N *et al.* Myeloablative allogeneic hematopoietic stem cell transplantation for non-Hodgkin lymphoma: a nationwide survey in Japan. *Blood* 108(1), 382-389 (2006).
9. Rodriguez J, Munsell M, Yazji S *et al.* Impact of high-dose chemotherapy on peripheral T-cell lymphomas. *J Clin Oncol* 19(17), 3766-3770 (2001).
10. Corradini P, Dodero A, Zallio F *et al.* Graft-versus-lymphoma effect in relapsed peripheral T-cell non-Hodgkin's lymphomas after reduced-intensity conditioning followed by allogeneic transplantation of hematopoietic cells. *J Clin Oncol* 22(11), 2172-2176 (2004).
11. Wulf Gg, Hasenkamp J, Jung W, Chapuy B, Truemper L, Glass B. Reduced intensity conditioning and allogeneic stem cell transplantation after salvage therapy integrating alemtuzumab for patients with relapsed peripheral T-cell non-Hodgkin's lymphoma. *Bone Marrow Transplant* 36(3), 271-273 (2005).
12. Corradini P. Vu, Rambaldi A., Miceli R., Patriarca F., Gallamini A. Intensified Chemo-Immunotherapy Including up-Front Autologous or Allogeneic Stem Cell Transplantation (SCT) for Young Patients with Newly Diagnosed Peripheral T-Cell Lymphomas: Final Results of a Phase II Multicenter Prospective Clinical Trial. *Blood* Abstract(120), 1984 (2012).
13. Piekarz Rl, Frye R, Turner M *et al.* Phase II multi-institutional trial of the histone deacetylase inhibitor Romidepsin as monotherapy for patients with cutaneous T-cell lymphoma. *J Clin Oncol* 27(32), 5410-5417 (2009).
14. Coiffier Bertrand, Pro Barbara, Prince H. Miles *et al.* Results from a pivotal, open-label, phase II study of Romidepsin in relapsed or refractory peripheral T-cell lymphoma after prior systemic therapy. J Clin Oncol.30(6): (2012).
15. Dupuis Jehan Cr-O, Herve Ghesquieres, Franck Morschhauser, Herve Tilly5, Catherine Thieblemont, Vincent Ribrag and Bertrand Coiffier. A Phase Ib Trial of Romidepsin in Association with CHOP in Patients with Peripheral T-Cell Lymphoma (PTCL): The Ro-CHOP Study. *Blood* 120(21)(2012).
16. Peart MJ, Smyth GK, van Laar RK, et al. Identification and functional significance of genes regulated by structurally different histone deacetylase inhibitors. Proc Natl Acad Sci USA 2005; 102:3697-3702.
17. Johnstone RW, Licht JD. Histone deacetylase inhibitors in cancer therapy: Is transcription the primary target? Cancer Cell 2003; 4:13-18.
18. Rasheed W, Bishton M, Johnstone RW, et al. Histone deacetylase inhibitors in lymphoma and solid malignancies. Expert Rev Anticancer Ther 2008; 8:413-432.
19. Bolden JE, Peart MJ, Johnstone RW. Anticancer activities of histone deacetylase inhibitors. Nat Rev Drug Discov 2006; 5:769-784.
20. Cress WD, Seto E. Histone deacetylases, transcriptional control, and cancer. J Cell Physiol 2000; 184:1-16.
21. Vigushin DM, Coombes RC. Histone deacetylase inhibitors in cancer treatment. Anticancer Drugs 2002; 13:1-13.
22. Santini V, Gozzini A, Ferrari G: Histone deacetylase inhibitors: Molecular and biological activity as a premise to clinical application. Curr Drug Metab 2007;8:383-393.

23. Whittaker SJ, Demierre MF, Kim EJ, et al. Final results from a multicenter, international, pivotal study of Romidepsin in refractory cutaneous T-cell lymphoma. J Clin Oncol 2010; 28:4485-4491.
24. O'Quigley J, Zohar S. Experimental designs for phase I and phase I/II dose-finding studies. Br J Cancer. 2006;94(5):609-13.
25. Zohar S, Chevret S. The continual reassessment method: comparison of Bayesian stopping rules for dose-ranging studies. Stat Med. 2001;20(19):2827-43.
26. Zohar S, Latouche A, Taconnet M, Chevret S. Software to compute and conduct sequential Bayesian phase I or II dose-ranging clinical trials with stopping rules. Comput Methods Programs Biomed. 2003;72(2):117-25.
27. O'Quigley J, Zohar S. Experimental designs for phase I and phase I/II dose-finding studies. Br J Cancer. 2006;94(5):609-13.
28. Case LD, Morgan TM. Design of Phase II cancer trials evaluating survivalprobabilities. BMC Med Res Methodol. 2003 Apr 3;3:6.
29. Zohar S, Chevret S. The continual reassessment method: comparison of Bayesian stopping rules for dose-ranging studies. Stat Med. 2001;20(19):2827-43.
30. Zohar S, Latouche A, Taconnet M, Chevret S. Software to compute and conduct sequential Bayesian phase I or II dose-ranging clinical trials with stopping rules. Comput Methods Programs Biomed. 2003;72(2):117-25.
31. Cheson BD, Fisher RI, Barrington SF, Cavalli F, Schwartz LH, Zucca E, and Lister TA. Recommendations for initial evaluation, staging, and response assessment of Hodgkin and Non-Hodgkin Lymphoma: the Lugano Classification. J Clin Oncol 2014; 32(27):3059-3067.
32. Barrington SF, Mikhaeel NG, Kostakoglu L, Meignan M, Hutchings M, Mu¨eller SP, Schwartz LH, Zucca E, Fisher RI, Trotman J, Hoekstra OS, Hicks RJ, O'Doherty MJ, Hustinx R, Biggi A, and Cheson BD. Role of imaging in the staging and response assessment of Lymphoma: Consensus of the International Conference on Malignant Lymphomas Imaging Working Group. J Clin Oncol 2014; 32(27):3048-58.

20 APPENDIXES

Appendix 1: Timing of treatment and investigations

Stage	Pre-Treatment	Ro-CHOEP Treatment				DHAP+SCT phase	Follow-Up
**Period**	**Screening**	**Cycle 1-2**	**Cycle 3**	**Cycle 4-5**	**Cycle 6**	**End of Treatment (+1 month)**	**Up to 2 years**
Informed consent	X						
Review Inclusion/Exclusion criteria	X						
Pregnancy test[a]	X						
Serum virology[b]	X						
Ecg [h]/Echo/MUGA	X [h]	X [h]	X [h]	X [h]	X [h]		
Clinical examination	X		X		X	X	X
Spirometry DLCO	X						
Bone marrow biopsy	X		X[c]		X[c]	X[c]	X[c]
Central pathological review	X						
PET scan[e]	X		X[i]		X	X	X[e]
CT of neck, chest, abdomen and pelvis	X		X		X	X	X
Adverse events		X [g]	X [g]	X [g]	X [g]	X [g]	X
Hematology	X	X [g]	X [g]	X [g]	X [g]	X [g]	X
Biochemistry[d]	X	X [g]	X [g]	X [g]	X [g]	X [g]	X
Physical examination	X	X [g]	X [g]	X [g]	X [g]	X [g]	X
Vital signs	X	X [g]	X [g]	X [g]	X [g]	X [g]	X
Lumbar puncture	X						
Neurological visit, RMN brain/spine, GI endoscopy, ORL visit[f]	X[f]						
Tumor tissue collection for biological studies	X				X		X Post treatment or at relapse
Bone marrow Blood Collection for Biological studies (Optional)[c]	X [c]						X [c] Post treatment or at relapse
Peripheral Blood collection for biological studies	X						X Post treatment or at relapse
HLA typing	X[i]		X[j]				

[a] Negative pregnancy test is required 1 week before treatment for both pre-menopausal women and women who are <2 years after onset of menopause
[b] HBsAg, HBcAb, HCV and HIV serology
[c] Bone marrow biopsy should be repeated after two cycles and one month after the end of therapy only if positive at screening. Biopsies during follow-up for patients who never had bone marrow involvement, or who became negative after treatment are to be performed only if clinically indicated at the discretion of the physician (same approach will be followed for gastrointestinal involvement).
[d] Biochemistry should include LDH and Beta2-microglobulin at screening
[e] When clinically indicated
[f] According to physician judge when clinically relevant for the patient
[g] During treatment stage, hematology tests will be performed at least once a week, while physical examination, biochemistry and adverse events monitoring will be performed every 2 weeks.
[h] Ecg for measurement of corrected QT interval according to the Fridericia formula
[i] Not mandatory
[j] HLA typing and start donor search mandatory for patients in PR

Appendix 2: Response Criteria

Table 3. Revised Criteria for Response Assessment

Response and Site	PET-CT–Based Response	CT-Based Response
Complete	Complete metabolic response	Complete radiologic response (all of the following)
Lymph nodes and extralymphatic sites	Score 1, 2, or 3* with or without a residual mass on 5PS†<br>It is recognized that in Waldeyer's ring or extranodal sites with high physiologic uptake or with activation within spleen or marrow (eg, with chemotherapy or myeloid colony-stimulating factors), uptake may be greater than normal mediastinum and/or liver. In this circumstance, complete metabolic response may be inferred if uptake at sites of initial involvement is no greater than surrounding normal tissue even if the tissue has high physiologic uptake	Target nodes/nodal masses must regress to ≤ 1.5 cm in LDi<br>No extralymphatic sites of disease
Nonmeasured lesion	Not applicable	Absent
Organ enlargement	Not applicable	Regress to normal
New lesions	None	None
Bone marrow	No evidence of FDG-avid disease in marrow	Normal by morphology; if indeterminate, IHC negative
Partial	Partial metabolic response	Partial remission (all of the following)
Lymph nodes and extralymphatic sites	Score 4 or 5† with reduced uptake compared with baseline and residual mass(es) of any size<br>At interim, these findings suggest responding disease<br>At end of treatment, these findings indicate residual disease	≥ 50% decrease in SPD of up to 6 target measurable nodes and extranodal sites<br>When a lesion is too small to measure on CT, assign 5 mm × 5 mm as the default value<br>When no longer visible, 0 × 0 mm<br>For a node > 5 mm × 5 mm, but smaller than normal, use actual measurement for calculation
Nonmeasured lesions	Not applicable	Absent/normal, regressed, but no increase
Organ enlargement	Not applicable	Spleen must have regressed by > 50% in length beyond normal
New lesions	None	None
Bone marrow	Residual uptake higher than uptake in normal marrow but reduced compared with baseline (diffuse uptake compatible with reactive changes from chemotherapy allowed). If there are persistent focal changes in the marrow in the context of a nodal response, consideration should be given to further evaluation with MRI or biopsy or an interval scan	Not applicable
No response or stable disease	No metabolic response	Stable disease
Target nodes/nodal masses, extranodal lesions	Score 4 or 5 with no significant change in FDG uptake from baseline at interim or end of treatment	< 50% decrease from baseline in SPD of up to 6 dominant, measurable nodes and extranodal sites; no criteria for progressive disease are met
Nonmeasured lesions	Not applicable	No increase consistent with progression
Organ enlargement	Not applicable	No increase consistent with progression
New lesions	None	None
Bone marrow	No change from baseline	Not applicable
Progressive disease	Progressive metabolic disease	Progressive disease requires at least 1 of the following
Individual target nodes/nodal masses	Score 4 or 5 with an increase in intensity of uptake from baseline and/or	PPD progression:
Extranodal lesions	New FDG-avid foci consistent with lymphoma at interim or end-of-treatment assessment	An individual node/lesion must be abnormal with:<br>LDi > 1.5 cm and<br>Increase by ≥ 50% from PPD nadir and<br>An increase in LDi or SDi from nadir<br>0.5 cm for lesions ≤ 2 cm<br>1.0 cm for lesions > 2 cm<br>In the setting of splenomegaly, the splenic length must increase by > 50% of the extent of its prior increase beyond baseline (eg, a 15-cm spleen must increase to > 16 cm). If no prior splenomegaly, must increase by at least 2 cm from baseline<br>New or recurrent splenomegaly
Nonmeasured lesions	None	New or clear progression of preexisting nonmeasured lesions
New lesions	New FDG-avid foci consistent with lymphoma rather than another etiology (eg, infection, inflammation). If uncertain regarding etiology of new lesions, biopsy or interval scan may be considered	Regrowth of previously resolved lesions<br>A new node > 1.5 cm in any axis<br>A new extranodal site > 1.0 cm in any axis; if < 1.0 cm in any axis, its presence must be unequivocal and must be attributable to lymphoma<br>Assessable disease of any size unequivocally attributable to lymphoma
Bone marrow	New or recurrent FDG-avid foci	New or recurrent involvement

Abbreviations: 5PS, 5-point scale; CT, computed tomography; FDG, fluorodeoxyglucose; IHC, immunohistochemistry; LDi, longest transverse diameter of a lesion; MRI, magnetic resonance imaging; PET, positron emission tomography; PPD, cross product of the LDi and perpendicular diameter; SDi, shortest axis perpendicular to the LDi; SPD, sum of the product of the perpendicular diameters for multiple lesions.

*A score of 3 in many patients indicates a good prognosis with standard treatment, especially if at the time of an interim scan. However, in trials involving PET where de-escalation is investigated, it may be preferable to consider a score of 3 as inadequate response (to avoid undertreatment). Measured dominant lesions: Up to six of the largest dominant nodes, nodal masses, and extranodal lesions selected to be clearly measurable in two diameters. Nodes should preferably be from disparate regions of the body and should include, where applicable, mediastinal and retroperitoneal areas. Non-nodal lesions include those in solid organs (eg, liver, spleen, kidneys, lungs), GI involvement, cutaneous lesions, or those noted on palpation. Nonmeasured lesions: Any disease not selected as measured, dominant disease and truly assessable disease should be considered not measured. These sites include any nodes, nodal masses, and extranodal sites not selected as dominant or measurable or that do not meet the requirements for measurability but are still considered abnormal, as well as truly assessable disease, which is any site of suspected disease that would be difficult to follow quantitatively with measurement, including pleural effusions, ascites, bone lesions, leptomeningeal disease, abdominal masses, and other lesions that cannot be confirmed and followed by imaging. In Waldeyer's ring or in extranodal sites (eg, GI tract, liver, bone marrow), FDG uptake may be greater than in the mediastinum with complete metabolic response, but should be no higher than surrounding normal physiologic uptake (eg, with marrow activation as a result of chemotherapy or myeloid growth factors).

†PET 5PS: 1, no uptake above background; 2, uptake ≤ mediastinum; 3, uptake > mediastinum but ≤ liver; 4, uptake moderately > liver; 5, uptake markedly higher than liver and/or new lesions; X, new areas of uptake unlikely to be related to lymphoma.

Appendix 3: NCI Common toxicity criteria

In the present study, adverse events and/or adverse drug reactions will be recorded according to the:
Common Terminology Criteria for Adverse Events (CTCA), version 4.0.
At the time this protocol was issued, the full CTC document was available on the NCI web site, at the following address: http://ctep.cancer.gov/reporting/ctc.html.

Appendix 4: Drug Preparation/Administration/Dispensing information for Romidepsin

Romidepsin for injection will be supplied by Celgene, as a kit containing two vials in a single carton, one vial of Romidepsin for injection and one vial of diluent. The drug vial will contain a lyophilized powder of 10 mg of Romidepsin and 20 mg of povidone, USP (used as a bulking agent). The diluent vial will contain 2 mL (deliverable volume) of a 4:1 mixture of propylene glycol and ethanol. The carton must be stored at 20° to 25°C. The vials containing the investigational product and the kits they are packaged in will be labeled according to the Good Manufacturing Practice guidelines and the local requirements with regard to the Clinical Trials. The Sponsor is responsible for checking that all information reported in the label is compliant with the Annex 13. All drug packages are to be inspected upon receipt at the study site prior to patient use. Romidepsin should be reconstituted by appropriately trained personnel using an aseptic technique. If any particulate matter is detected, the kit is not to be used. Damaged kits are to be reported to the Manufacturer and stored until instructions have been given.
The appropriate amount of Romidepsin will be calculated at each treatment administration based on the body surface area (BSA) of each individual patient. The dual pack is to be stored at 20 to 25ºC, excursions permitted between 15 to 30°C [USP controlled room temperature]. Romidepsin (for infusion) is stable for at least 36 months at 25°C/60% relative humidity (RH) as well as for 6 months at 40°C/75% RH and is stable against heat (for 3 months at 50°C) and humidity (for 3 months at 25°C/83% RH). The drug should be reconstituted by appropriately trained personnel using aseptic technique. A volume of 2 mL of reconstitution diluent is added to the lyophilized powder and swirled until contents of each vial are free from visible particles. The number of vials to be reconstituted should be determined according to the dose to be administered and the patient BSA. The reconstituted product stock solution at 5 mg/mL is chemically stable for at least 8 hours at room temperature. However, whenever possible, drug should be prepared within 4 hours of dose administration. The volume of the 5 mg/mL stock solution containing the appropriate dose for the patient will be diluted in 0.9% Sodium Chloride Injection, USP (0.9% saline) for intravenous infusion, as directed by the protocol. This dilution should result in a final drug concentration within the demonstrated stability range of 0.02 to 0.16 mg/mL for reconstituted Romidepsin, that is compatible with polyvinyl chloride (PVC), ethylene vinyl acetate (EVA), and polyethylene (PE) intravenous infusion bags; glass bottles may also be used. The Romidepsin infusion solution is chemically stable for at least 24 hours at room temperature. However, whenever possible, drug should be prepared within 4 hours of dose administration.

Appendix 5: Suggested Body Surface Area Calculation

BSA should be determined using the appropriate following calculation:

$$BSA = \sqrt{\frac{Ht(inches) \times Wt(lbs)}{3131}}$$

OR

$$BSA = \sqrt{\frac{Ht(cm) \times Wt(kg)}{3600}}$$

Appendix 6: Creatinine Clearance Calculation

Creatinine clearance for men and women will be calculated according to the Cockcroft-Gault formula as follows:

In men:

$$\frac{[(140 - age) \times weight\ (kg)]}{[72 \times creatinine\ (mg / dL)]}$$

In women:

$$\frac{[(140 - age) \times weight\ (kg)]}{[72 \times creatinine\ (mg / dL)]} \times 0.85$$

Note: Age (in years), weight (in kg), serum-creatinine (in mg/dL)
72 (normalized to 72 kg body weight and a body surface of 1.72 m^2)

Appendix 7: ECOG performance status scale

Grade	Description
0	Fully active, able to carry on all pre-disease performance without restriction
1	Restricted in physically strenuous activity but ambulatory and able to carry out work of a light or sedentary nature, e.g., light housework or office work
2	Ambulatory and capable of all self-care but unable to carry out any work activities. Up and about □□50% of waking hours
3	Capable of only limited self-care, confined to a bed or chair □□50% of waking hours
4	Completely disabled. Cannot carry on any self-care. Totally confined to bed or chair
5	Dead

Appendix 8: New York Heart Association Classification of Cardiac Disease

The following table presents the NYHA classification of cardiac disease:

Class	Functional Capacity	Objective Assessment
I	Patients with cardiac disease but without resulting limitations of physical activity. Ordinary physical activity does not cause undue fatigue, palpitation, dyspnea, or anginal pain.	No objective evidence of cardiovascular disease.
II	Patients with cardiac disease resulting in slight limitation of physical activity. They are comfortable at rest. Ordinary physical activity results in fatigue, palpitation, dyspnea, or anginal pain.	Objective evidence of minimal cardiovascular disease.
III	Patients with cardiac disease resulting in marked limitation of physical activity. They are comfortable at rest. Less than ordinary activity causes fatigue, palpitation, dyspnea, or anginal pain.	Objective evidence of moderately severe cardiovascular disease.
IV	Patients with cardiac disease resulting in inability to carry on any physical activity without discomfort. Symptoms of heart failure or the anginal syndrome may be present even at rest. If any physical activity is undertaken, discomfort is increased.	Objective evidence of severe cardiovascular disease.

Source: The Criteria Committee of the New York Heart Association, Inc.: Diseases of the heart and blood vessels; Nomenclature and criteria for diagnosis, 6th Ed. Boston: Little, Brown; 1964.

Appendix 9: The Hematopoietic Cell Transplant-Comorbidity Index (HCT-CI)

Patient __________________________________ ***(name), UPN*** _____________ ***Date*** ____________

Comorbidities	Definitions	HCT-CI weighted scores	Actual Lab Values/Comments
Arrhythmia	Atrial fibrillation or flutter, sick sinus syndrome, and ventricular arrhythmias	1	
Cardiac	Coronary artery disease†, congestive heart failure, myocardial infarction, or EF≤50%	1	
Inflammatory bowel disease	Crohn's disease or ulcerative colitis	1	
Diabetes*	Requiring treatment with insulin or oral hypoglycemic, but not diet alone	1	
Cerebro-vascular disease	Transient ischemic attack or cerebro-vascular accident	1	
Psychiatric disturbance*	Depression/anxiety requiring psychiatric consult or treatment	1	
Hepatic – mild*	Chronic hepatitis, Bilirubin >ULN- 1.5 X ULN, or AST/ALT >ULN-2.5XULN	1	
Obesity*	Patients with a body mass index >35 kg/m^2	1	
Infection*	Requiring continuation of anti-microbial treatment after day 0	1	
Rheumatologic	SLE, RA, polymyositis, mixed CTD, polymyalgia rheumatica	2	
Peptic ulcer	Requiring treatment	2	
Moderate/severe renal*	Serum creatinine >2 mg/dl, on dialysis, or prior renal transplantation	2	
Moderate pulmonary*	DLco and/or FEV_1 >65%-80% or Dyspnea on slight activity	2	
Prior solid tumor	Treated at any time point in the patient's past history, excluding non-melanoma skin cancer	3	
Heart valve disease*	Except mitral valve prolapse	3	
Severe pulmonary*	DLco and/or FEV_1 ≤65% or Dyspnea at rest or requiring oxygen	3	
Moderate/severe hepatic*	Liver cirrhosis, Bilirubin >1.5 X ULN, or AST/ALT >2.5XULN	3	
		Total Score =____________	

* Comorbidity is currently active or patient requires medical treatment.

†One or more vessel-coronary artery stenosis, requiring medical treatment, stent, or bypass graft.

EF indicates ejection fraction; ULN, upper limit of normal; SLE, systemic lupus erythmatosis; RA, rheumatoid arthritis; CTD, connective tissue disease; DLco, diffusion capacity of carbon monoxide; FEV_1, forced expiratory volume in one second; AST, aspartate aminotransferase; ALT, alanine aminotransferase.

Appendix 10: Grading of Acute Graft-Versus-Host Disease

Severity of Individual Organ Involvement		
Skin	+1	a maculopapular eruption involving less than 25% of the body surface
	+2	a maculopapular eruption involving 25-50% of the body surface
	+3	generalized erythroderma
	+4	generalized erythroderma with bullous formation and often with desquamation
Liver	+1	bilirubin (2.0-3.0 mg/100 ml)
	+2	bilirubin (3-5.9 mg/100 ml)
	+3	bilirubin (6-14.9 mg/100 ml)
	+4	bilirubin > 15 mg/100 ml
Gut	Diarrhea is graded +1 to +4 in severity. Nausea and vomiting and/or anorexia caused by GVHD is assigned as +1 in severity<br>The severity of gut involvement is assigned to the most severe involvement noted. Patients with visible bloody diarrhea are at least stage +2 gut and grade +3 overall	
Diarrhea	+1	≤ 1000 ml of liquid stool/day* (≤ 15ml of stool/kg/day)†
	+2	>1,000 ml of stool/day* (> 15ml of stool/kg/day)†
	+3	>1,500 ml of stool/day* (> 20ml of stool/kg/day)†
	+4	2,000 ml of stool/day* (≥ 25ml of stool/kg/day)†

**In the absence of infectious/medical cause*

†For pediatric patients
